# Tuning mesophase topology in hydrogen-bonded liquid crystals *via* halogen and alkyl chain engineering

**DOI:** 10.1039/d5ra08676k

**Published:** 2026-01-05

**Authors:** Ahmed F. Darweesh, Christian Anders, Mohamed Alaasar

**Affiliations:** a Department of Chemistry, Faculty of Science, Cairo University 12613 Giza Egypt; b Institute of Chemistry, Martin Luther University Halle-Wittenberg 06120 Halle Germany mohamed.alaasar@chemie.uni-halle.de

## Abstract

This study explores the influence of halogen substitution and alkyl chain length on the liquid crystalline properties of hydrogen-bonded supramolecules. Three series of hydrogen-bonded liquid crystals (HBLCs) were synthesized by combining 4-alkoxyphenylazopyridines as proton acceptors with varying alkyl chain lengths and *ortho*-halogenated (F, Cl, or Br) 4-dodecyloxybenzoic acids as proton donors. The formation of hydrogen-bonding interactions between the individual components was confirmed using FTIR spectroscopy. The self-assembly behavior of these HBLCs was characterized using differential scanning calorimetry (DSC), polarized optical microscopy (POM), and X-ray diffraction (XRD). Our findings demonstrate that systematic variation of the halogen atom and alkyl chain length profoundly impacts mesophase stability and type. Specifically, fluorinated HBLCs exhibit elevated melting and clearing temperatures, whereas their chlorinated and brominated counterparts show lower melting points and broader mesophase ranges. The choice of halogen also determines the type of liquid crystalline phases, resulting in the formation of tilted smectic C (SmC), orthogonal smectic A (SmA), and nematic phases. Furthermore, these materials exhibit rapid and reversible *trans–cis* photoisomerization upon light exposure. This work elucidates design principles for tuning the properties of HBLCs through synergistic halogen and chain-length engineering.

## Abstract 2

Halogen and alkyl chain engineering of hydrogen-bonded liquid crystals. Halogen- and chain-dependent mesophase behaviour and reversible photo-switching.

## Graphical abstract

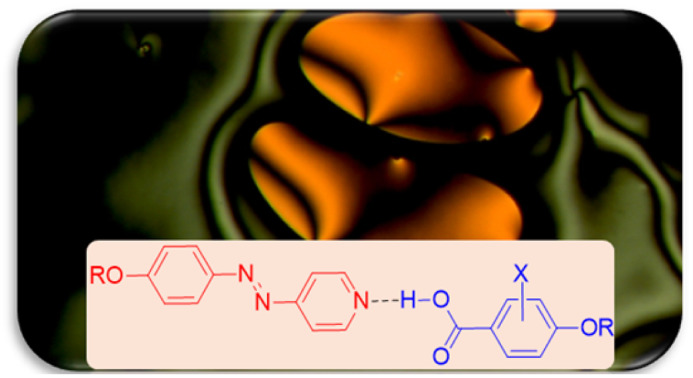

## 1. Introduction

Liquid crystals (LCs) are a fascinating class of materials that combine the long-range order of crystals with the fluidity of liquids. Their unique ability to self-assemble into various ordered mesophases and respond to external stimuli makes them indispensable for a wide array of applications, including tunable optical filters,^1^ variable-focus lenses,^2^ biosensors,^3,4^ optical switches,^5^ smart windows,^6^ holographic patterns,^7^ drug delivery systems,^8^ and many other applications.

Photoresponsive materials are a cornerstone of functional soft matter, engineered to undergo precise and reversible changes in their properties upon light irradiation.^9^ The absorption of light acts as a remote, non-invasive trigger, inducing molecular-scale phenomena such as isomerization, cyclization, or bond cleavage. This primary effect can profoundly alter intermolecular interactions, leading to a programmed reorganization of self-assembled superstructures.^10–15^ Among the most well-known photoresponsive units is azobenzene, which exhibits a reversible *trans–cis* photoisomerization.^16^ The rod-like, thermodynamically stable *trans* isomer can be switched to a bent *cis* configuration under UV light,^17^ a property that is highly effective in modifying the liquid crystalline order.^18–20^ An even more versatile building block for supramolecular LCs is azopyridine.^21,22^ The nitrogen atom in the pyridine ring renders azopyridine derivatives excellent hydrogen or halogen bond acceptors. This enables the construction of complex, stimuli-responsive liquid crystalline architectures from simpler, and even non-mesogenic, building blocks *via* highly directional non-covalent interactions.^21,23–27^

The strength and directionality of hydrogen bonds are highly sensitive to the chemical environment, including the presence of substituents that can alter electron density.^18,28–30^ Therefore, the presence of halogen substituents can impact hydrogen bonding by altering the acidity of hydrogen bond donors or the basicity of acceptors through inductive and resonance effects. The polarizability of halogen substituents can further modulate the intermolecular interactions by increasing London dispersion forces and dipole–dipole interactions. This dual effect allows for precise control over mesophase stability, transition temperatures, and material responsiveness.

Fluorine is one of the most prevalent lateral substituents in liquid crystal design.^31,32^ Its high electronegativity, small steric effect (Immirzi crystal volume, cv ∼13 nm^3^),^33^ and low polarizability allow for precise modification of mesophase behaviour without suppressing liquid crystallinity. This has enabled the tuning of LC behaviour in diverse systems, from rod-like^34^ and bent-core mesogens^35–37^ to the recent induction of ferroelectricity in nematic phases of calamitic LCs.^38,39^ Beyond fluorine, other halogens such as chlorine,^40–45^ with cv ∼27 nm^3^ and bromine,^46–51^ with cv ∼33 nm^3^ can also be employed. However, their larger atomic volumes compared to fluorine impart more pronounced steric effects, leading to more significant alterations in molecular packing and mesophase morphology.

**Fig. 1:**
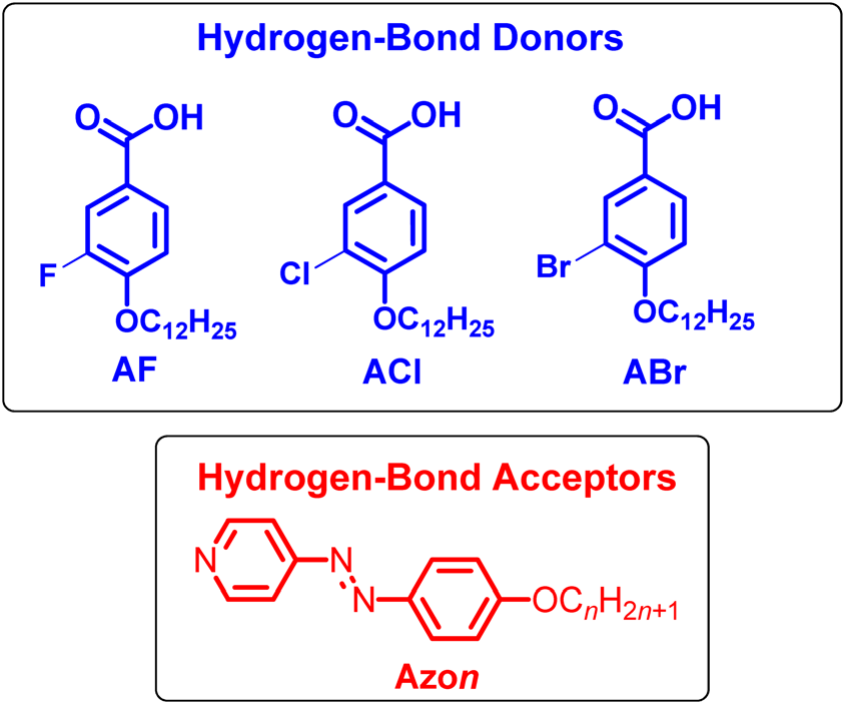
Chemical structures of hydrogen-bond donors and hydrogen-bond acceptors under discussion.

While our recent work has explored the influence of aromatic core fluorination in HBLCs,^22^ a systematic comparative study of different halogens remains lacking. Herein, we report the design and characterization of new HBLCs to systematically investigate the impact of fluorine, chlorine, and bromine substituents on their liquid crystalline properties. Therefore, three different groups of HBLCs were designed and prepared (Fig. 1) in which 4-alkoxyphenylazopyridines (Azo*n*) with a variable terminal chain (*n* = 8, 10, 12, 14) were used as proton acceptors. The proton donors are 3-halogenated-4-dodecyloxybenzoic acids (AX), having a fixed terminal chain (OC_12_H_25_) and X = F, Cl, and Br. Through a combination of differential scanning calorimetry (DSC), polarized optical microscopy (POM), and X-ray diffraction (XRD), we establish clear structure–property relationships in such HBLCs. Additionally, the incorporation of an azopyridine unit enables reversible *trans–cis* photoisomerization, underscoring the potential of these materials for photonic applications.

## 2. Synthesis

### 2.1. Synthesis of proton donors and proton acceptors

**Scheme 1:**
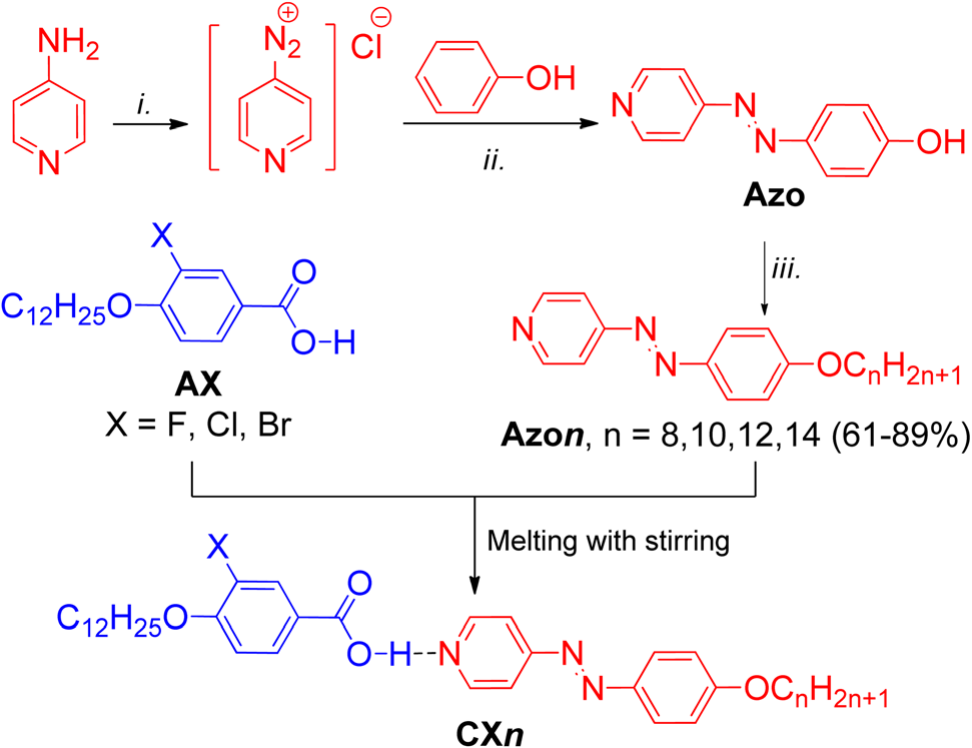
Synthesis of the new HBLCs CX*n*. Reagents and conditions: (i) NaNO_2_/HCl/H_2_O, 0 °C, 5 h; (ii) NaOH, NaHCO_3_; (iii) BrC_*n*_H_2*n*+1_, K_2_CO_3_, DMF, 80 °C, 18 h.

The synthesis of the new HBLCs CX*n* was performed as shown in Scheme 1. The azopyridine derivatives Azo*n* were synthesized using previously reported methods,^23,52,53^ while the synthesis of the proton donors AX was carried out according to the procedures described in ref. 54 (for X = F and Br) and that in ref. 55 (for X = Cl). The general synthetic procedures and analytical data for the synthesized azopyridines and benzoic acid derivatives are provided in the SI.

### 2.2. Preparation of HBCLs supramolecules (CX*n*)

The target supramolecules CX*n* were prepared by heating equimolar (1 : 1) mixtures of acid AX and the corresponding azopyridine Azo*n* until a homogeneous melt formed. After cooling to ambient temperature, the resulting orange solids were subjected to a second melting cycle to ensure complete complexation. The reproducible phase transition temperatures and homogeneous melting observed in DSC measurements confirm the formation of stable hydrogen-bonded complexes.

## 3. Results and discussion

### 3.1. FTIR studies

**Fig. 2:**
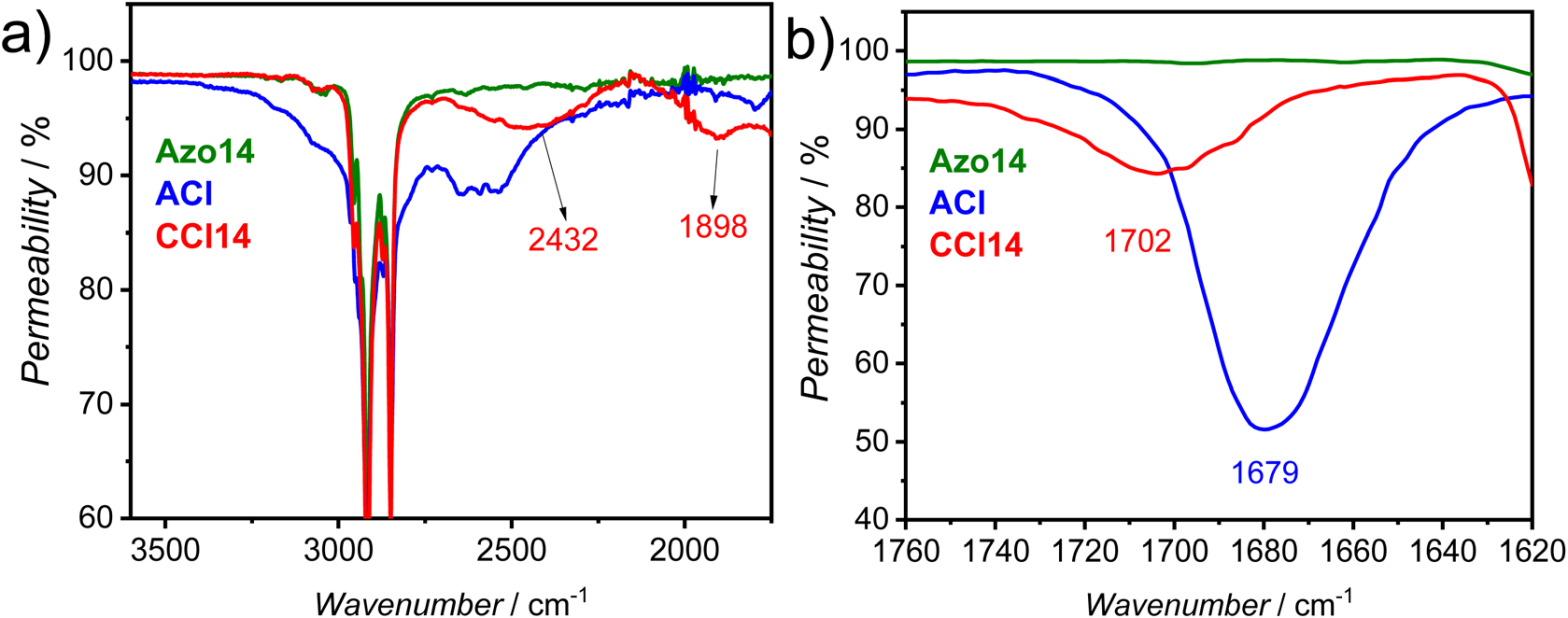
FTIR spectra of the supramolecule CCl14 (red) and its complementary components the acid ACl (blue) and the azopyridine Azo14 (green) in the crystalline state at room temperature, enlarged area: (a) between 1750 cm^−1^ and 3600 cm^−1^, and (b) between 1620 cm^−1^ and 1760 cm^−1^.

The formation of the hydrogen-bonded supramolecular complexes was confirmed *via* Fourier-transform infrared (FTIR) spectroscopy, performed using the KBr pellet method for a selected representative example from each group of the HBLCs. Therefore, we chose for such investigations the supramolecules with *n* = 14, *i.e.*, CF14, CCl14, and CBr14. The FTIR spectra of the supramolecule CCl14 and its individual components ACl and Azo14 are given in Fig. 2a and b, while those of CF14 and CBr14 are displayed in Fig. S8 and S9, respectively.

The FTIR spectrum of CCl14 in Fig. 2 provides clear evidence of hydrogen bond formation. A direct comparison with the spectra of its complementary components, the 3-chloro-4-dodecylbenzoic acid ACl and the azopyridine Azo14, reveals significant spectral changes. The characteristic broad O–H stretching band of the carboxylic acid ACl, which is initially present in a dimer form before complexation with Azo14, observed between 2500 and 3300 cm^−1^ is notably absent in the complex CCl14. Instead, two new, broad bands emerge at approximately 2432 and 1898 cm^−1^ (Fig. 2a), which are attributed to Fermi resonance overtones characteristic of a hydrogen-bonded O–H⋯N pair.^22,28^

Further evidence is found in the carbonyl stretching region (Fig. 2b). The *ν*_C=O_ band of the pure acid ACl at 1679 cm^−1^ undergoes a distinct shift to a higher wavenumber (∼1702 cm^−1^) upon complexation, accompanied by band broadening and a decrease in intensity. This shift is consistent with a change in the carbonyl environment upon disruption of the acid dimer (–COOH⋯HOOC–) and formation of the new COO–H⋯N hydrogen bond. Collectively, these spectroscopic features agree with those reported for other benzoic acid derivatives–pyridine-based supramolecular systems,^22,56^ thereby confirming the formation of the target HBLCs. Similar results were obtained for the supramolecules CF14 and CBr14 (Fig. S8 and S9), further confirming the successful formation of hydrogen-bonding interactions in all cases.

### 3.2. DSC and POM investigations

**Fig. 3:**
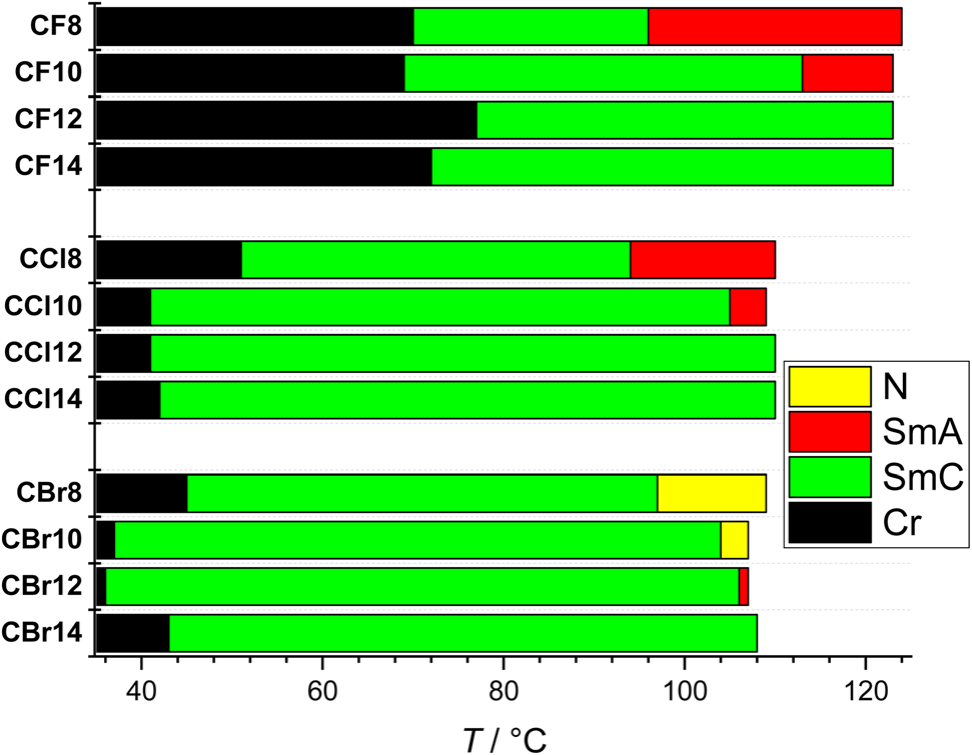
Bar diagram indicating the different examined LC phases of the various hydrogen-bonded supramolecules CF*n*, CCl*n*, and CBr*n* as recorded on cooling.

Table 1 summarizes the transition temperatures, associated enthalpies, and liquid crystal phase types of the prepared HBLCs as obtained from DSC and POM investigations. The results are also represented graphically in Fig. 3. The data reveal a clear dependence of the mesophase behaviour on both the nature of the halogen substituent and the alkyl chain length (*n*). It should be noted here that all the azopyridines (Azo*n*)^22^ are crystalline materials and do not exhibit any LC phase. On the other hand, the halogenated benzoic acids melt directly into an isotropic liquid state on heating, and on cooling, the chlorinated acid (ACl) does not show any LC, while the brominated and fluorinated acids (ABr and AF) show a small range of smectic A (SmA) and smectic C, respectively (see Fig. S10–S12 for DSC). However, all the targeted hydrogen-bonded materials are liquid crystalline materials with different types of LC phases and relatively wide temperature ranges (Table 1 and Fig. 3), revealing the importance of hydrogen bonding interaction for the induction of mesomorphism.

**Table 1:** Liquid crystal phases, transition temperatures, and transition enthalpies of the supramolecules CX*n*^a^

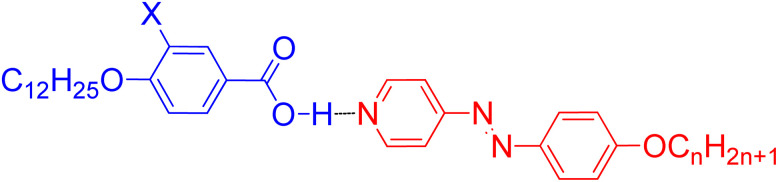		
CX*n*	*n*	*T/*°C [Δ*H*/kJ mol^−1^]
CF8	8	H: Cr 88 [39.8] SmC_s_ 98 [—] SmA 129 [12.3] Iso
		C: Iso 124 [−11.7] SmA 96 [—] SmC_s_ 70 [−40.3] Cr
CF10	10	H: Cr 80 [28.1] SmC_s_ 115 [—] SmA 128 [9.1] Iso
		C: Iso 123 [−8.6] SmA 113 [—] SmC_s_ 69 [−11.0] Cr
CF12	12	H: Cr 89 [30.0] SmC_s_ 126 [10.2] Iso
		C: Iso 123 [−9.1] SmC_s_ 77 [−29.3] Cr
CF14	14	H: Cr 91 [41.1] SmC_s_ 127 [16.1] Iso
		C: Iso 123 [−14.8] SmC_s_ 72 [−39.7] Cr
CCl8	8	H: Cr 76 [58.4] SmC_s_ 96 [—] SmA 115 [8.0] Iso
		C: Iso 110 [−7.4] SmA 94 [—] SmC_s_ 51 [−33.1] Cr
CCl10	10	H: Cr 64 [68.6] SmC_s_ 106 [—] SmA 114 [12.4] Iso
		C: Iso 109 [−10.5] SmA 105 [—] SmC_s_ 41 [−57.5] Cr
CCl12	12	H: Cr 70 [22.1] SmC_s_ 114 [12.8] Iso
		C: Iso 110 [−14.4] SmC_s_ 41 [−17.1] Cr
CCl14	14	H: Cr 82 [47.7] SmC_s_ 114 [12.8] Iso
		C: Iso 110 [−13.6] SmC_s_ 42 [−36.2] Cr
CBr8	8	H: Cr 84 [78.1] SmC_s_ 102 [0.4] N 115 [9.3] Iso
		C: Iso 109 [−8.9] N 97 [−0.5] SmC_s_ 45 [−60.0] Cr
CBr10	10	H: Cr 65 [53.9] SmC_s_ 104 [—] N 110 [9.9] Iso
		C: Iso 107 [−9.3] N 104 [—] SmC_s_ 37 [−43.4] Cr
CBr12	12	H: Cr 74 [40.3] SmC_s_ 109 [—] SmA 111 [12.4] Iso
		C: Iso 107 [−12.9] SmA 106 [—] SmC_s_ 36 [−43.8] Cr
CBr14	14	H: Cr 87 [48.6] SmC_s_ 112 [13.5] Iso
		C: Iso 108 [0.13.7] SmC_s_ 43 [36.7] Cr

a Peak temperatures as determined by DSC (10 K min^−1^) on heating (H) and cooling (C); Cr: crystalline solid; SmC_s_: synclinic smectic C phase; SmA: smectic A phase; N: nematic phase; Iso: isotropic liquid.

#### 3.2.1. Fluorinated HBLCs (CF*n*)

Depending on the length of the terminal chain *n* at the azopyridine side, the fluorinated supramolecules CF*n* exhibit different types of LC phases. Therefore, CF8 and CF10 display two LC phases, while the longer homologues with *n* ≥ 12 display only one phase. Notably, the fluorinated HBLCs generally show higher clearing temperatures *T*_c_ (LC-Iso transitions on heating) compared to their chlorinated (CCl*n*) and brominated (CBr*n*) counterparts with similar alkyl chain lengths (Table 1 and Fig. 3). For instance, CF8 has *T*_c_ ∼126 °C, while that of both CCl8 and CBr8 is ∼115 °C. This suggests that the strong electronegativity of fluorine leads to increased molecular polarity. This polarity, in conjunction with its minimal steric demand, enhances the overall intermolecular cohesion, which contributes to the elevated transition temperatures and thermal stability of the fluorinated HBLCs compared to their chlorinated and brominated analogues.

**Fig. 4:**
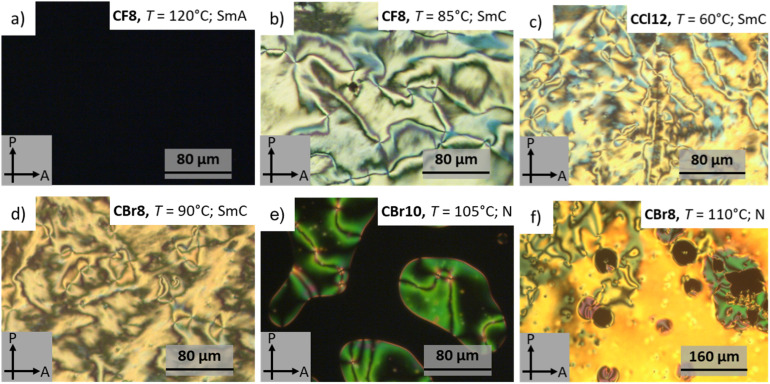
Optical textures observed on cooling for representative examples of HBLCs in different LC phases at the indicated temperatures.

**Fig. 5:**
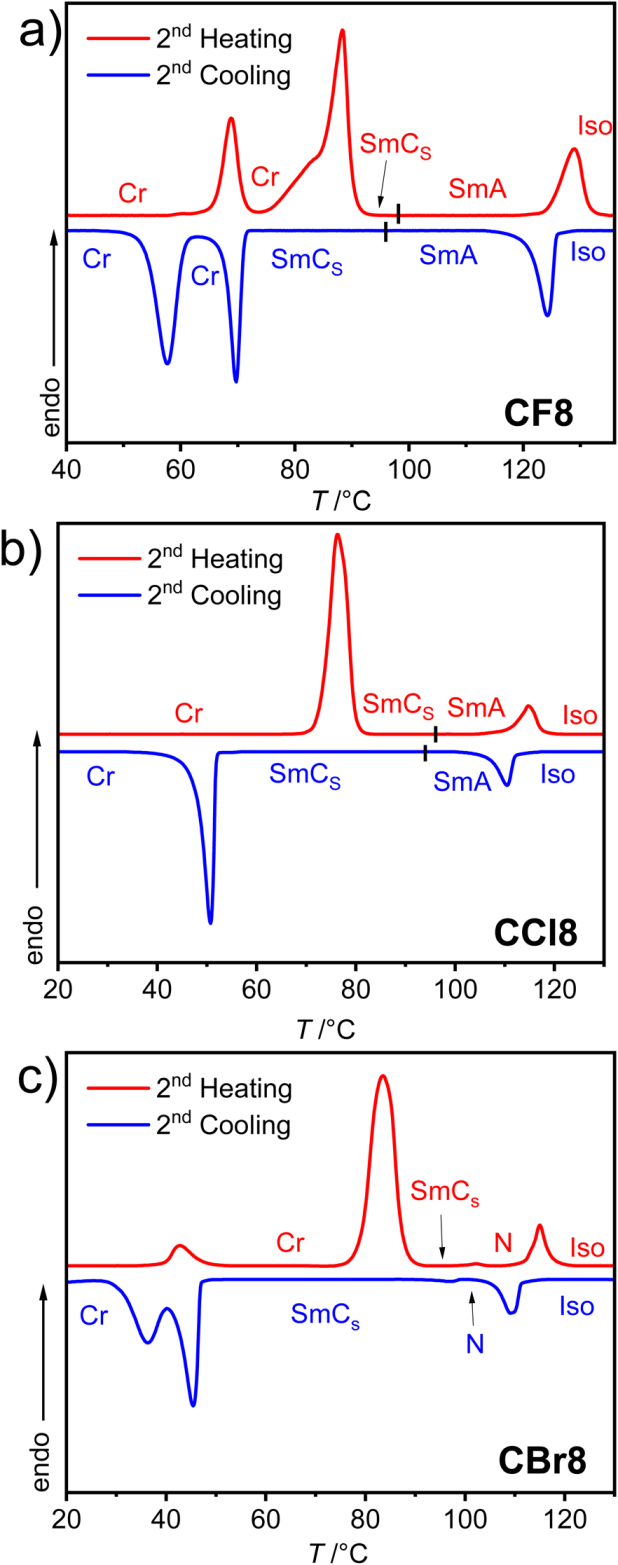
DSC heating and cooling traces of the supramolecules: (a) CF8; (b) CCl8, and (c) CBr8 recorded at 10 K min^−1^.

Under POM, the different types of LC phases could be easily identified. As an example, Fig. 4a shows the optical textures observed for CF8 on cooling from the isotropic liquid phase. In the higher temperature LC phase at *T* ∼120 °C, a totally dark texture could be seen, which is associated with a transition peak in the DSC heating scan (Fig. 5a and Table 1). This LC is fluid but more viscous compared to a normal isotropic liquid, which is a characteristic feature of an orthogonal non-titled lamellar smectic A (SmA) phase. On further cooling of the SmA phase and at *T* ∼96 °C, a highly birefringent texture is observed, confirming the onset of molecular tilt and the transition to another LC phase (Fig. 4b).

This phase transition cannot be detected by DSC but is observed only under POM, indicating a second-order transition. As shown in Fig. 4b, the lower-temperature LC is characterized by four-brush disclination, which is typically observed for synclinic smectic C (SmC_s_) LC phases.^57,58^ Based on these POM observations, the phase sequence of CF8 is designated as Iso–SmA–SmC_s_–Cr on cooling. The type of LC phases was further confirmed by XRD studies (see Section 3.3). The POM investigations of CF8 on heating confirmed the presence of Cr–SmC_s_–SmA–Iso phase transitions, meaning that the smectic phases of CF8 are enantiotropic ones. The same phase sequence is also exhibited by the next homologue CF10, but with a narrower range of SmA phase. For the longer derivatives CF12 and CF14, the SmA phase is completely absent, and only SmC is observed (Fig. 3).

#### 3.2.2. Chlorinated HBLCs (CCl*n*)

The chlorinated series (CCl*n*) also displays SmA and SmC_s_ phases like the CF*n* series (Fig. 4c and 5b). Therefore, the two shorter homologues exhibit the enantiotropic phase sequence SmC_s_–SmA but with a shorter range of SmA phase compared to CF8 and CF10. The larger size of the chlorine atom compared to the fluorine one results in a significant reduction of the melting and crystallization temperatures in the CCl*n* series (Table 1). This leads to wider LC phase ranges for all members of CCl*n* series. For instance, the longer homologue CCl14 has a SmC_s_ range of ∼68 K on cooling, while its related fluorinated analogue CF14 has a narrower range of ∼51 K of the same phase.

#### 3.2.3. Brominated HBLCs (CBr*n*)

The third group of HBLCs, CBr*n*, incorporates the largest halogen substituent used in this work, *i.e.*, bromine. In contrast to the fluorinated and chlorinated analogues, the brominated series exhibits richer polymorphism, including nematic (N), SmA, and SmC_s_ phases. Notably, the shortest homologue, CBr8, displays a nematic phase over a relatively short range (∼13 K on heating), unlike the SmA phase observed for CF8 and CCl8. This nematic range further narrows for CBr10 (∼6 K). The nematic phase was identified by its characteristic Schlieren texture, which flashes under mechanical shear (Fig. 4e and f). The emergence of the nematic phase in the brominated (CBr*n*) series suggests that the significant steric bulk and high polarizability of the bromine atom weaken intermolecular interactions relative to the fluorine and chlorine analogues. This favors the formation of the less-ordered nematic phase over the more structured SmA phase.

Elongating the chain to *n* = 12 in CBr12 eliminates the nematic phase, yielding a short-range SmA phase followed by the SmC_s_ phase. For the longest homologue (CBr14), the SmA phase is entirely suppressed, leaving the SmC_s_ phase as the only mesophase – a trend mirrored in the other series (Fig. 3). A key consequence of the large bromine substituent is the significantly wider LC phase range compared to the chlorinated analogues (CCl*n*). While both series (CCl*n* and CBr*n*) exhibit similar clearing temperatures, the greater steric demand of bromine suppresses crystallization more effectively, resulting in the lowest crystallization temperatures among all HBLCs (Table 1). This results in the broadest LC phase ranges for the CBr*n* series without suppressing mesomorphism (Fig. 3c).

The evolution of the mesophase sequence across the CBr*n* series – from N for short chains (*n* = 8, 10) to a short-range SmA phase for *n* = 12, and finally to a stable SmC_s_ phase for *n* = 14 – can be rationalized by considering the balance between the overall molecular aspect ratio and the local steric perturbation of the bromine substituent. For the shorter homologues, the large van der Waals volume of the bromine atom constitutes a significant proportional deviation from a perfect rod-like shape, frustrating the formation of layered smectic order and favouring the less ordered nematic phase. As the alkyl chain length increases, the overall molecular length and thus its aspect ratio increase significantly. Consequently, the steric disruption caused by the bromine atom becomes a relatively smaller perturbation to the molecular shape. This shift allows the stronger van der Waals interactions between the longer alkyl chains and the improved molecular shape anisotropy to dominate, progressively stabilizing smectic phases (SmA and SmC_s_). This underscores that the mesophase morphology is not governed by the absolute steric bulk of the substituent alone, but by its effect relative to the entire molecular dimension.

The mesomorphic behaviour across all three series correlates directly with the halogen's electronegativity and size. Fluorine, being the smallest and most electronegative, enhances intermolecular interactions *via* strong inductive effects, leading to higher transition temperatures. Its minimal steric bulk also facilitates compact packing, stabilizing the SmA phase in CF8 and CF10. In contrast, chlorine and bromine exhibit lower electronegativity but greater size and polarizability. This shifts the balance towards wider LC ranges and suppresses the SmA phase. The appearance of nematic phases in shorter brominated homologues indicates that steric hindrance from the bulky bromine atom disrupts the formation of smectic layers, favouring a less ordered nematic arrangement. For CBr12, this hindrance is partially overcome by the longer alkyl chain, allowing a short-range SmA phase to emerge above the SmC_s_ phase. Additionally, within each series, increasing the alkyl chain length (*n*) generally influences the mesophase stability and transition temperatures (Table 1). Longer alkyl chains typically promote smectic phases due to increased van der Waals interactions between the chains; therefore, only SmC_s_ phase is observed for the longest member in each series of the HBLCs.

### 3.3 X-ray diffraction (XRD) investigations

**Fig. 6:**
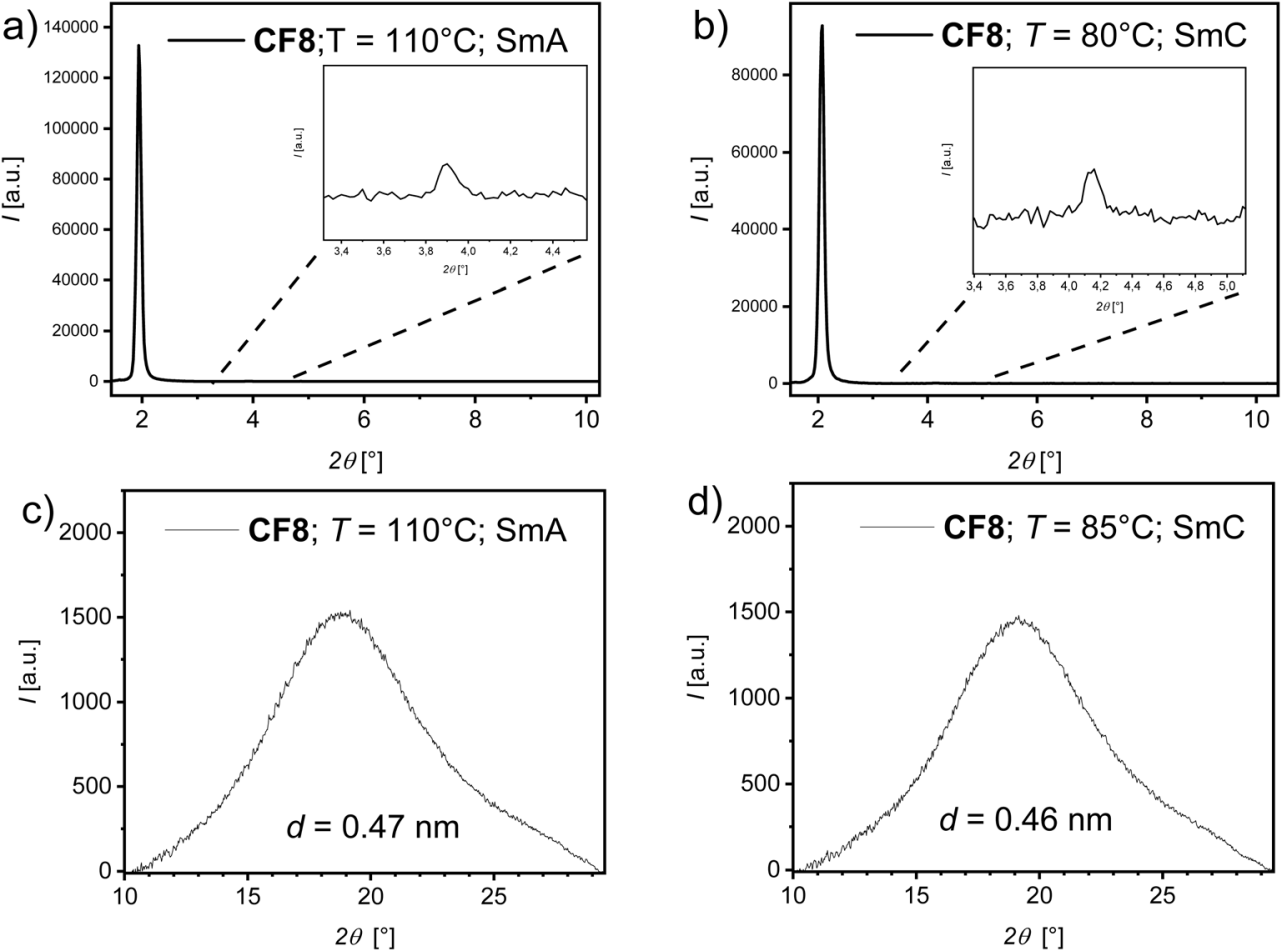
XRD patterns recorded on cooling with a cooling rate of 10 K min^−1^ of the supramolecule CF8 in the different LC phases at the indicated temperatures: (a) and (b) SAXS patterns; (c) and (d) WAXS patterns.

To confirm the type of smectic LC phases observed for the prepared HBLCs, X-ray diffraction (XRD) studies were performed on representative supramolecules, CF8, CCl8, and CBr12, in their mesophases. Temperature-dependent small-angle X-ray scattering (SAXS) and wide-angle X-ray scattering (WAXS) measurements provided detailed information about the long-range molecular organization and short-range intermolecular correlations, respectively. The analysis of the diffraction patterns obtained for CF8 at 110 °C for the higher temperature LC phase and at 80 °C for the lower temperature one allowed for the identification of the two distinct mesophases as SmA and SmC phases. The SAXS pattern for the SmA phase, recorded at 110 °C, is characterized by a sharp, intense reflection at 2*θ*° ∼2.07 with a second-order small reflection at 2*θ*° ∼4.14 in the small-angle region (Fig. 6a). The reciprocal spacing of this primary reflection corresponds to a *d*-spacing of ∼4.54 nm, which is equivalent to the molecular length (*L*) of the supramolecular complex CF8, estimated to be approximately 4.54 nm from Materials Studio.

Concurrently, the WAXS pattern (Fig. 6c) exhibits a diffuse, broad halo in the wide-angle region centered at approximately *d* = 0.47 nm. This halo is characteristic of the average lateral separation between the molten, disordered aliphatic chains. The combination of a sharp small-angle reflection and a diffuse wide-angle halo is a definitive signature of a smectic A mesophase. In this phase, the molecules are arranged in layers with the long molecular axis, on average, perpendicular to the layer plane. These observations agree with the optical texture recorded for CF8 in the SmA phase (Fig. 4a).

Upon cooling to 85 °C, the WAXS pattern (Fig. 6d) continues to show a diffuse halo, confirming the retention of a liquid crystal phase. The SAXS pattern (Fig. 6b) reveals a measurable decrease in the layer spacing to *d* = 4.27 nm. This contraction is a classic indicator of a transition from a SmA to a SmC phase, where the molecules tilt at a finite angle (*θ*) with respect to the layer normal, which also agrees with increased birefringence observed under POM at the SmA–SmC transition (Fig. 4b). Similar XRD results were obtained for the supramolecules CCl8 and CBr12 (see Section 3 in the SI).

## 4. Optical properties and photo-responsive behaviour

The photophysical and photo-responsive properties of the HBLCs were investigated to elucidate the effect of the halogen atom (F, Cl and Br) on their behaviour under light irradiation. UV-vis spectroscopy was used to monitor spectral changes in chloroform solution, while POM was employed to study light-induced phenomena in the LC phases.

### 4.1. UV-vis spectroscopy and photo-isomerization in solution

**Fig. 7:**
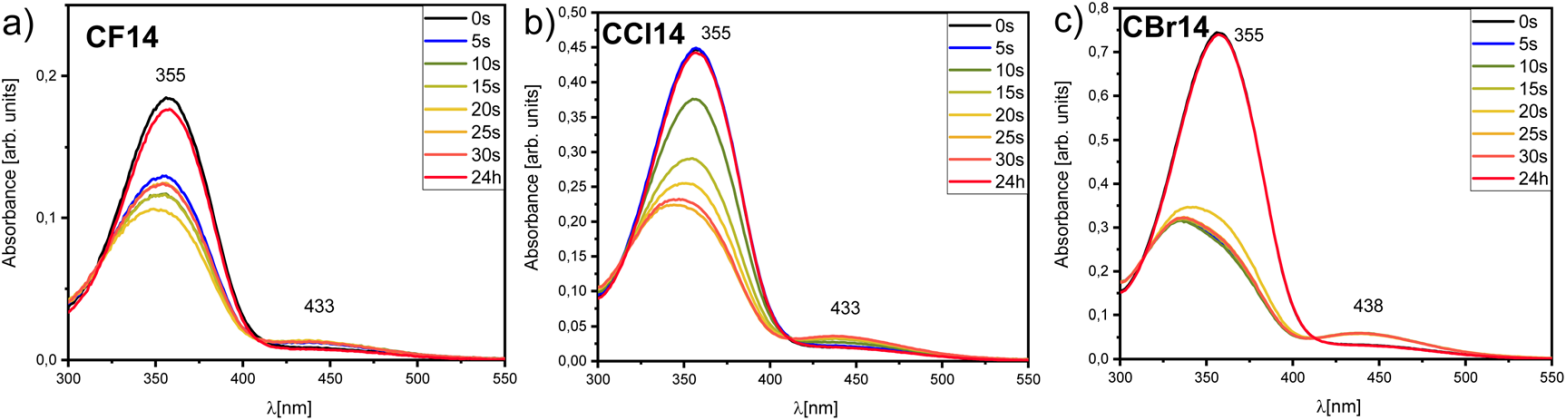
Spectral changes observed under UV light irradiation for: (a) CF14, (b) CCl14, and (c) CBr14.

The photo-responsive nature of the supramolecular complexes CF14, CCl14, and CBr14 with the same terminal chains was probed in chloroform solution by monitoring their UV-vis spectra under UV light irradiation (Fig. 7). All three materials exhibited a strong π–π* transition band in the UV region, characteristic of the photoactive core (the azopyridine derivative). Upon irradiation, a distinct decrease in the intensity of this primary absorption band was observed for all materials, indicative of a photo-isomerization process. This is consistent with a *trans* to *cis* isomerization of the azo unit incorporated within the supramolecular structure. The rate and extent of this spectral change were found to be halogen-dependent. The bromine-substituted complex CBr14 (Fig. 7c) exhibited the most pronounced and rapid decrease in absorbance, followed by CFl14 (Fig. 7a) and then CCl14 (Fig. 7b). All materials exhibit reversible *cis–trans* photoisomerization as indicated by the very similar spectra obtained in all cases after keeping the solution in the dark overnight.

### 4.2. Photo-induced phase transitions in the liquid crystalline state

**Fig. 8:**
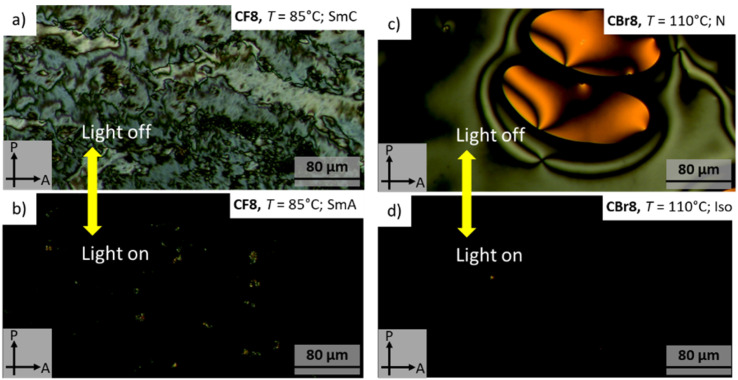
Isothermal photo-induced reversible LC phase transitions observed under POM upon light irradiation for: (a), (b) SmA–SmC transition in CF8 at 85 °C, and (c), (d) N-Iso transition in CBr8 at 110 °C.

The photo-responsive behaviour of CF8 and CBr8, as representative examples, was studied using POM under UV light irradiation at different temperatures, corresponding to various LC phases. In the SmC phase of CF8 at 85 °C, irradiation with UV light caused a transition to the upper temperature SmA (Fig. 8a and b). When the light was switched off, the original texture recovered, demonstrating the fully reversible nature of this effect. In the nematic phase of CBr8 at 110 °C, UV light triggered a transition to the isotropic liquid (Iso) phase (Fig. 8c and d). This transition was also reversible, with the birefringent nematic texture reforming upon removal of the UV stimulus. The reversible photo-switching process in both cases was very fast and took place in less than three seconds.

The observed behaviour across all phases can be attributed to the light-induced shape change from a linear *trans*-isomer to a bent *cis*-isomer. This molecular-level shape changes effectively “dopes” the system with a non-mesogenic molecule, disrupting the delicate balance of interactions that stabilize the liquid crystalline order. These results demonstrate that these halogenated H-bonded supramolecules are highly efficient photo-switches. The ability to precisely and reversibly induce textural changes and even isothermal phase transitions with light makes them outstanding candidates for advanced applications in photo-active soft materials, such as optical memory devices, light-driven actuators, and re-writable photonic systems.^9,59^

## 5. Summary and conclusion

This study systematically investigated the effect of halogen substituents (F, Cl, Br) on the liquid crystalline behaviour of hydrogen-bonded supramolecules. Our findings demonstrate that the nature of the halogen atom significantly influences the phase morphology, transition temperatures, and thermal stability. Fluorinated derivatives generally exhibit higher clearing temperatures and a preference for smectic phases, attributed to fluorine's strong electronegativity and small size, which enhance intermolecular interactions. In contrast, brominated supramolecules show a greater tendency toward nematic phases, particularly with shorter alkyl chains (*n* = 8 and 10), likely due to the larger size and higher polarizability of bromine, which can alter molecular packing and the balance of intermolecular forces. For these shorter brominated derivatives, the significant steric volume of bromine relative to the overall molecular length strongly perturbs the rod-like shape, preventing layered smectic order. However, upon elongation of the terminal alkyl chain (*n* ≥ 12), the increased molecular aspect ratio diminishes the relative impact of the halogen's steric bulk, allowing smectic phases to emerge. Between the fluorinated and brominated HBLCs, the chlorinated ones exhibit intermediate behaviour with the absence of nematic phases and the appearance of a short range of SmA phases for the shorter derivatives, thereby completing the halogen-dependent trend. These observations underscore that the halogen atom acts as a key modulator, fine-tuning the delicate balance between dipolar interactions and steric demands within the supramolecular architecture. Furthermore, these materials exhibit robust and reversible photo-switching in both solution and the liquid crystalline state. This dual-state responsiveness highlights their significant potential for advanced applications in functional soft matter, such as light-driven actuators, optical switches, and reconfigurable photonic devices.

In summary, this work provides a definitive framework for the rational design of supramolecular liquid crystals. By strategic selection of the halogen substituent and alkyl chain length, the mesomorphic properties can be precisely engineered, paving the way for a new generation of tuneable and functional materials.

## Conflicts of interest

There are no conflicts to declare.

## Supplementary Material

RA-016-D5RA08676K-s001

## Data Availability

The data supporting this article have been included as part of the supplementary information (SI). Supplementary information is available. See DOI: https://doi.org/10.1039/d5ra08676k.
