# Supplementary material for: Tuning mesophase topology in hydrogen-bonded liquid crystals via halogen and alkyl chain engineering
Source: RSC Adv. 2026 Jan 5;16(2):1556–64. doi: 10.1039/d5ra08676k (PMC12767301; doi:10.1039/d5ra08676k)
Supplement: RA-016-D5RA08676K-s001 [file RA-016-D5RA08676K-s001.pdf]

Supplementary Information (SI) for RSC Advances.
This journal is © The Royal Society of Chemistry 2025

Supporting Information

Tuning Mesophase Topology in Hydrogen-Bonded Liquid Crystals via Halogen and Alkyl Chain Engineering

Ahmed F. Darweesh,[a] Christian Anders,[b] Mohamed Alaasar*[b]

[a]Department of Chemistry, Faculty of Science, Cairo University, 12613 Giza, Egypt
[b]Institute of Chemistry, Martin Luther University Halle-Wittenberg, 06120 Halle, Germany

Contents

1. **Experimental**
2. **Additional Data**
3. **XRD Data**
4. **References**

1. Experimental

1.1. Characterization

Chemicals of analytical purity, sourced from commercial suppliers, were utilized as received. Solvents, when required, underwent drying using conventional techniques. Various spectral data were employed to confirm the purity and chemical composition of all synthesized compounds. Structure characterization of the produced materials was assessed by utilizing ^{1}H NMR, ^{13}C NMR, and ^{19}F NMR with Varian Unity 400 spectrometers in $CDCl_3$ solution. ^{1}H NMR and ^{13}C NMR chemical shifts are reported in ppm referenced to tetramethylsilane. The residual proton signal of the deuterated solvent was used as internal standard for ^{1}H NMR and ^{13}C NMR spectra. ^{19}F NMR chemical shifts are reported in ppm referenced to trichlorofluoromethane as an external standard.

Infrared absorption spectra were measured in dry KBr with a Perkin-Elmer B25 spectrophotometer.

The assessment of mesophase phases and the determination of transition temperatures for the hydrogen-bonded compounds involved using a Nikon Optiphot-2 polarizing microscope in conjunction with a Mettler FP-82 HT hot stage and control unit. Enthalpies were determined by analyzing DSC thermograms obtained with a Perkin-Elmer DSC-7 instrument, employing a heating and cooling rate of 10 K min^{-1}.

The photoisomerization studies in solution were conducted using an Ocean Optics HR 2000+ spectrophotometer, and absorption spectra were recorded at room temperature. The solutions in chloroform were taken in a 1cm quartz cuvette and covered to avoid the evaporation of the solvent. The solutions were irradiated with UV light of 1mW/cm^2 using Bluepoint LED Eco Hönle at a wavelength of 365 nm. A heat filter is inserted between the sample and the source to avoid the influence of UV heat on the sample. The *trans-cis-trans* photoisomerization in the LC phase was performed using of 1 mW/cm^2 Bluepoint LED Eco Hönle at a wavelength of 365 nm.

X-ray investigations were carried out with an Incoatec (Geesthacht, Germany) IμS microfocus source with a monochromator for CuKα radiation (λ = 0.154 nm), calibration with the powder pattern of $Pb(NO_3)_2$. A droplet of the sample was placed on a glass plate on a Linkam hot stage HFS-X350-GI (rate: 10 K/min). The samples were cooled from the isotropic liquid and held at specific temperatures in the respective LC phases during the individual XRD scans. Exposure time was 5 min; the sample-detector distance was 9.00 cm for WAXS and 26.80 cm for SAXS. The diffraction patterns were recorded with a Vantec 500 area detector (Bruker AXS, Karlsruhe) and transformed into 1D plots using GADDS software.

1.2. Synthesis of AX

The proton donors **AX** were synthesized starting from different key intermediates using standard reported procedures.[1,2]

4-(Dodecyloxy)-3-fluorobenzoic acid **AF**. ^{1}H NMR (402 MHz, cdcl$_3$) δ 7.87 (d, *J* = 8.7 Hz, 1H, ArH), 7.80 (d, *J* = 11.5, 1H, ArH), 6.99 (t, *J* = 8.3 Hz, 1H, ArH), 4.10 (t, *J* = 6.5 Hz, 2H, OCH_2), 1.87 – 1.82 (m, 2H, OCH_2*CH_2*), 1.52 – 1.18 (m, 18H, CH_2), 0.88 (t, *J* = 6.6 Hz, 3H, CH_3). ^{13}C NMR (101 MHz, cdcl$_3$) δ 170.28, 153.02, 152.14, 127.36, 121.50, 117.85, 113.30, 69.39, 31.87, 29.60, 29.58, 29.52, 29.47, 29.30, 29.26, 28.94, 25.81, 22.64, 14.06. ^{19}F NMR (378 MHz, cdcl$_3$) δ -133.84 (dd, *J* = 10.7, 8.8 Hz).

3-Chloro-4-(dodecyloxy)benzoic acid **ACl**. ^{1}H NMR (402 MHz, cdcl$_3$) δ 8.12 (d, *J* = 2.0 Hz, 1H, ArH), 7.98 (dd, *J* = 8.6, 2.0 Hz, 1H, ArH), 6.95 (d, *J* = 8.7 Hz, 1H, ArH), 4.10 (t, *J* = 6.5 Hz, 2H, OCH_2), 1.94 – 1.80 (m, 2H, OCH_2*CH_2*), 1.55 – 1.21 (m, 18H, CH_2), 0.88 (t, *J* = 6.8

Hz, 3H, CH_3). ^{13}C NMR (101 MHz, cdcl$_3$) δ 170.68, 159.01, 132.23, 130.48, 122.94, 121.90, 112.03, 69.37, 31.87, 29.61, 29.59, 29.52, 29.48, 29.30, 29.24, 28.85, 25.84, 22.65, 14.07.

3-Bromo-4-(dodecyloxy)benzoic acid **ABr**. ^{1}H NMR (402 MHz, cdcl$_3$) δ 8.29 (d, J = 1.6 Hz, 1H, ArH), 8.02 (d, J = 8.4 Hz, 1H, ArH), 6.91 (d, J = 8.6 Hz, 1H, ArH), 4.10 (t, J = 6.3 Hz, 2H, OCH_2), 2.00 – 1.77 (m, 2H, $OCH_2\mathit{CH_2}$), 1.64 – 1.08 (m, 18H, CH_2), 0.88 (t, J = 6.4 Hz, 3H, CH_3). ^{13}C NMR (101 MHz, cdcl$_3$) δ 170.54, 159.83, 135.40, 131.21, 122.33, 111.97, 111.83, 69.45, 31.87, 29.61, 29.60, 29.52, 29.48, 29.31, 29.22, 28.84, 25.86, 22.65, 14.07.

1.3.1. Synthesis of **Azo**

The general procedure involved adding a solution of sodium nitrite (2.28 g, 33 mmol, 1.1 eq.) dissolved in 12 ml of water and 25 ml of a 10% potassium hydroxide aqueous solution to phenol (30 mmol, 1.0 eq.). The solution was cooled to -20 °C using an acetone/dry ice mixture. Subsequently, a cooled solution of 4-aminopyridine (3.30 g, 36 mmol, 1.2 eq.) dissolved in 10 ml of water and 16 ml of concentrated HCl was added dropwise under vigorous stirring over 90 minutes. The reaction temperature was maintained at around -15 °C throughout the process. After the complete addition, the reaction mixture was stirred for an additional 1 hour, followed by the addition of sodium bicarbonate until no effervescence was observed. The resulting solid material was filtered off, washed with distilled water, dried under vacuum, and used without further purification for the next step.

1.3.2. Synthesis of **Azo*n***

Dissolving 2 mmol (1.0 eq.) of the specific 4-hydroxyphenylazopyridine **Azo** in 25 ml of DMF, along with the appropriate 1-bromoalkanes (2.4 mmol, 1.2 eq.), K_2CO_3 (6 mmol, 3 eq.), and a catalytic amount of KI, the reaction was agitated for 18 hours at 90°C. After bringing the reaction mixture to room temperature, it was poured into 100 mL of deionized water, resulting in a suspension. The suspension was then extracted with ethyl acetate (3 x 50 mL). The mixed organic layers were washed with water and $NaHCO_3$, dried on anhydrous $MgSO_4$, and the solvent was removed under reduced pressure. The desired products were obtained from the crude material using column chromatography with ethyl acetate/*n*-hexane (2:8).

4-(4-Octyloxyphenylazo)pyridine, **Azo8**. Orange solid. 80% Yield, m.p. 71 °C. ^{1}H NMR (600 MHz, $CDCl_3$) δ 8.77 (d, J = 6.1 Hz, 2H, Ar-H), 7.95 (d, J = 9.0 Hz, 2H, Ar-H), 7.67 (d, J = 6.1 Hz, 2H, Ar-H), 7.02 (d, J = 9.0 Hz, 2H, Ar-H), 4.06 (t, J = 6.6 Hz, 2H, -$OC\mathit{H_2}CH_2$), 1.86–1.77 (m, 2H, -$OCH_2C\mathit{H_2}$), 1.56–1.20 (m, 10H, CH_2), 0.90 (t, J = 7.0 Hz, 3H, CH_3). ^{13}C NMR

(151 MHz, $CDCl_3$) δ 162.9, 157.5, 151.1, 146.7, 125.6, 116.1, 114.9, 68.5, 31.8, 29.3, 29.2, 29.1, 25.9, 22.6, 14.1.

4-(4-Decyloxyphenylazo)pyridine, **Azo10**. Orange solid. 65% Yield, m.p. 66 °C. ^{1}H NMR (600 MHz, $CDCl_3$) δ 8.77 (d, J = 6.1 Hz, 2H, Ar-H), 7.95 (d, J = 8.9 Hz, 2H, Ar-H), 7.67 (d, J = 6.2 Hz, 2H, Ar-H), 7.02 (d, J = 8.9 Hz, 2H, Ar-H), 4.06 (t, J = 6.6 Hz, 2H, -OCH_2CH_2), 1.87–1.78 (m, 2H, -OCH_2CH_2), 1.52–1.21 (m, 14H, CH_2), 0.89 (t, J = 7.0 Hz, 3H, CH_3). ^{13}C NMR (151 MHz, $CDCl_3$) δ 162.9, 157.5, 151.1, 146.7, 125.6, 116.1, 114.9, 68.5, 31.8, 29.6, 29.5, 29.3, 29.2, 29.1, 25.9, 22.6, 14.1.

4-(4-Dodecyloxyphenylazo)pyridine, **Azo12**. Orange solid. 61% Yield, m.p. 73 °C. ^{1}H NMR (600 MHz, $CDCl_3$) δ 8.77 (d, J = 6.1 Hz, 2H, Ar-H), 7.95 (d, J = 9.0 Hz, 2H, Ar-H), 7.67 (d, J = 6.1 Hz, 2H, Ar-H), 7.02 (d, J = 9.0 Hz, 2H, Ar-H), 4.06 (t, J = 6.6 Hz, 2H, -OCH_2CH_2), 1.87–1.78 (m, 2H, -OCH_2CH_2), 1.53–1.21 (m, 18H, CH_2), 0.88 (t, J = 7.0 Hz, 3H, CH_3).^{13}C NMR (151 MHz, $CDCl_3$) δ 162.9, 157.5, 151.0, 146.7, 125.6, 116.1, 114.9, 68.5, 31.9, 29.6, 29.6, 29.5, 29.5, 29.3, 29.2, 29.1, 25.9, 22.6, 14.1.

4-(4-Tetradeylcoxyphenylazo)pyridine, **Azo14**. Orange solid. 65% Yield, m.p. 69 °C. ^{1}H NMR (600 MHz, $CDCl_3$) δ 8.77 (d, J = 5.9 Hz, 2H, Ar-H), 7.95 (d, J = 9.0 Hz, 2H, Ar-H), 7.67 (d, J = 6.0 Hz, 2H, Ar-H), 7.02 (d, J = 9.0 Hz, 2H, Ar-H), 4.06 (t, J = 6.6 Hz, 2H, -OCH_2CH_2), 1.84–1.80 (m, 2H, -OCH_2CH_2), 1.56–1.12 (m, 22H, CH_2), 0.88 (t, J = 7.0 Hz, 3H, CH_3).^{13}C NMR (151 MHz, $CDCl_3$) δ 162.9, 157.5, 151.0, 146.7, 125.6, 116.1, 114.9, 68.5, 31.9, 29.7, 29.6, 29.6, 29.5, 29.5, 29.3, 29.1, 25.9, 22.6, 14.1.

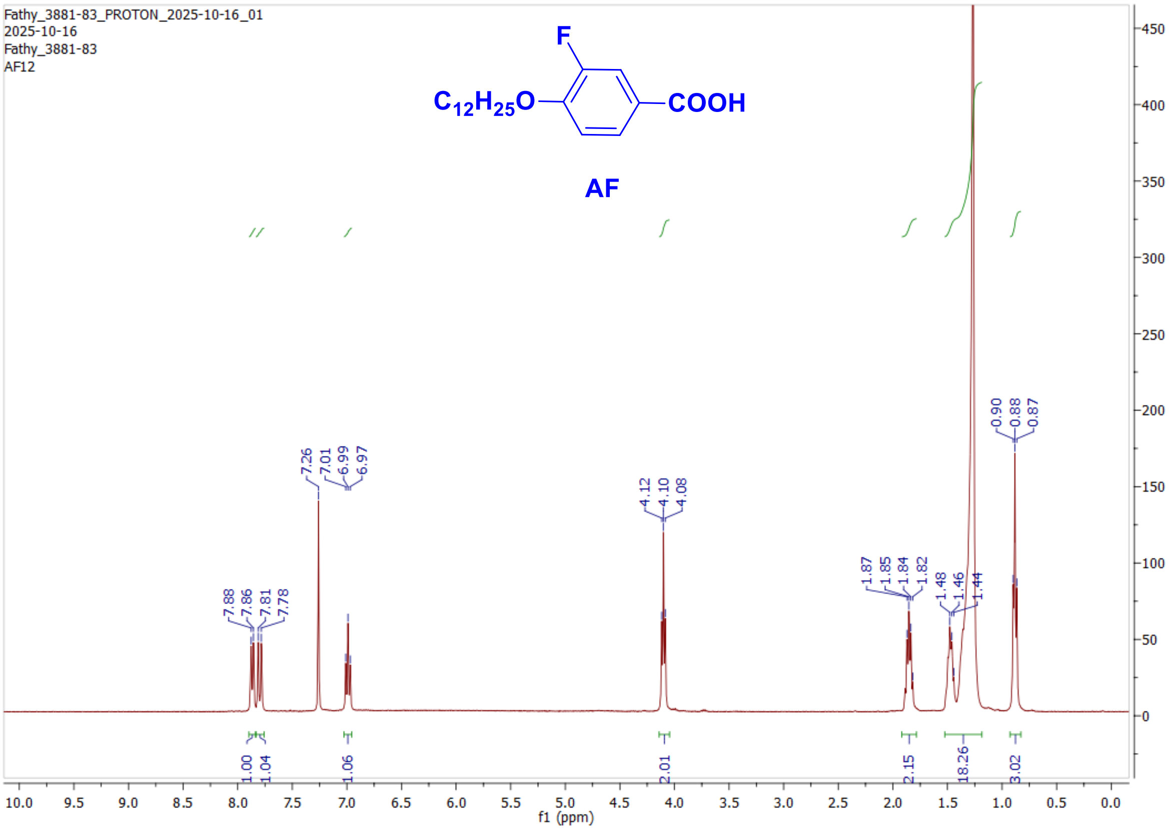

Figure S1a. ^{1}H NMR of compound **AF**

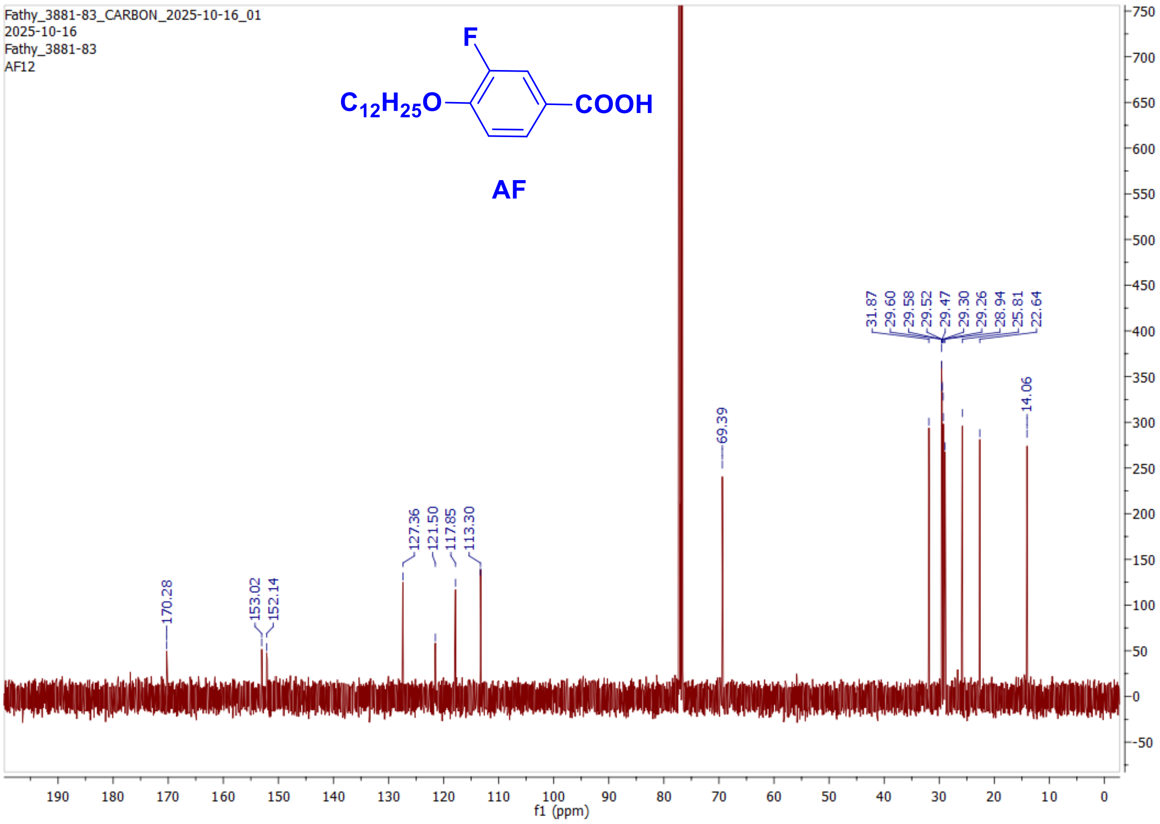

Figure S1b. ^{13}C NMR of compound **AF**

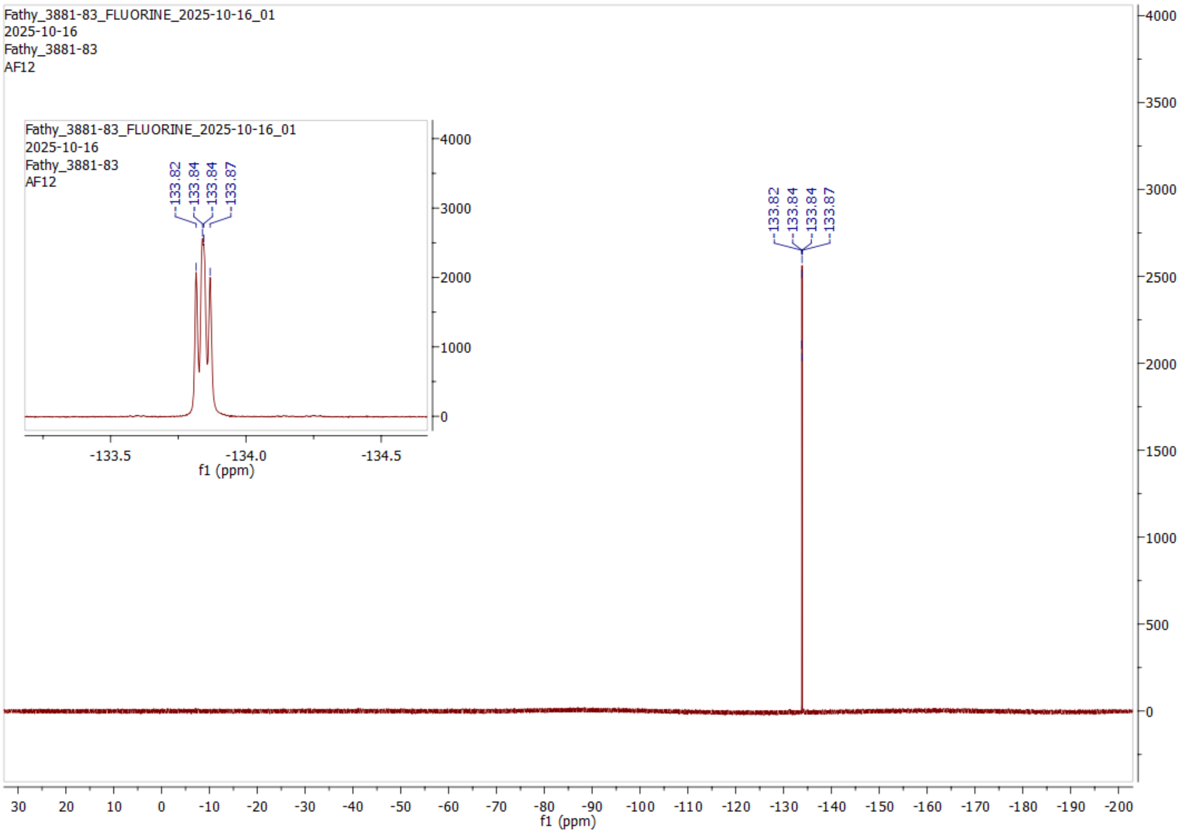

Figure S1c. ^{19}F NMR of compound **AF**

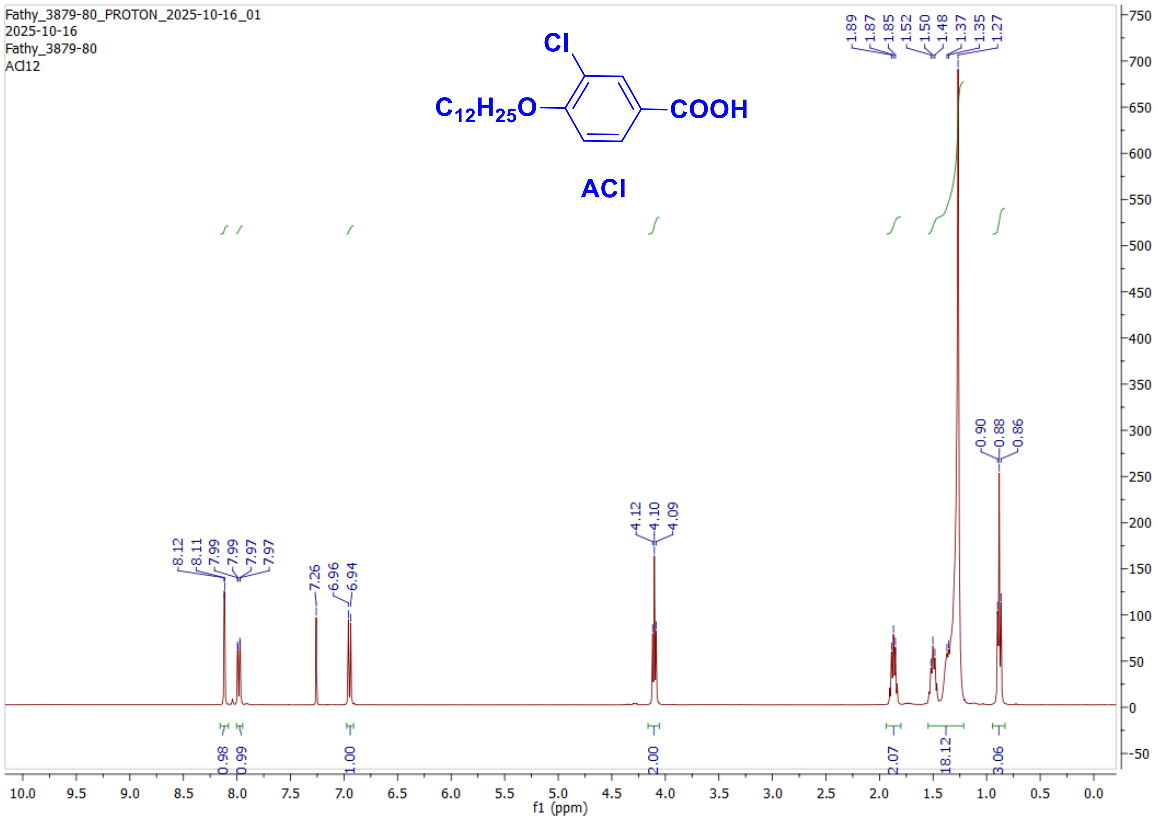

Figure S2a. ^{1}H NMR of compound **ACl**

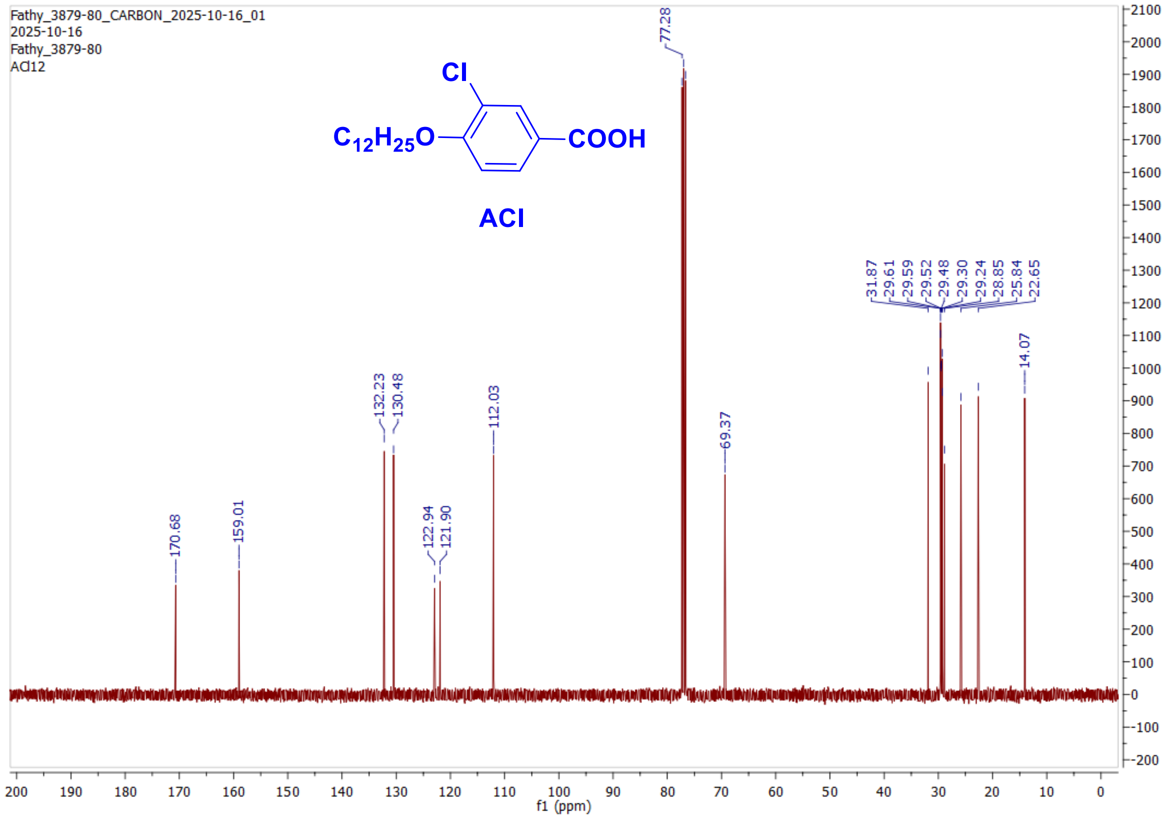

Figure S2b. ^{13}C NMR of compound **ACl**

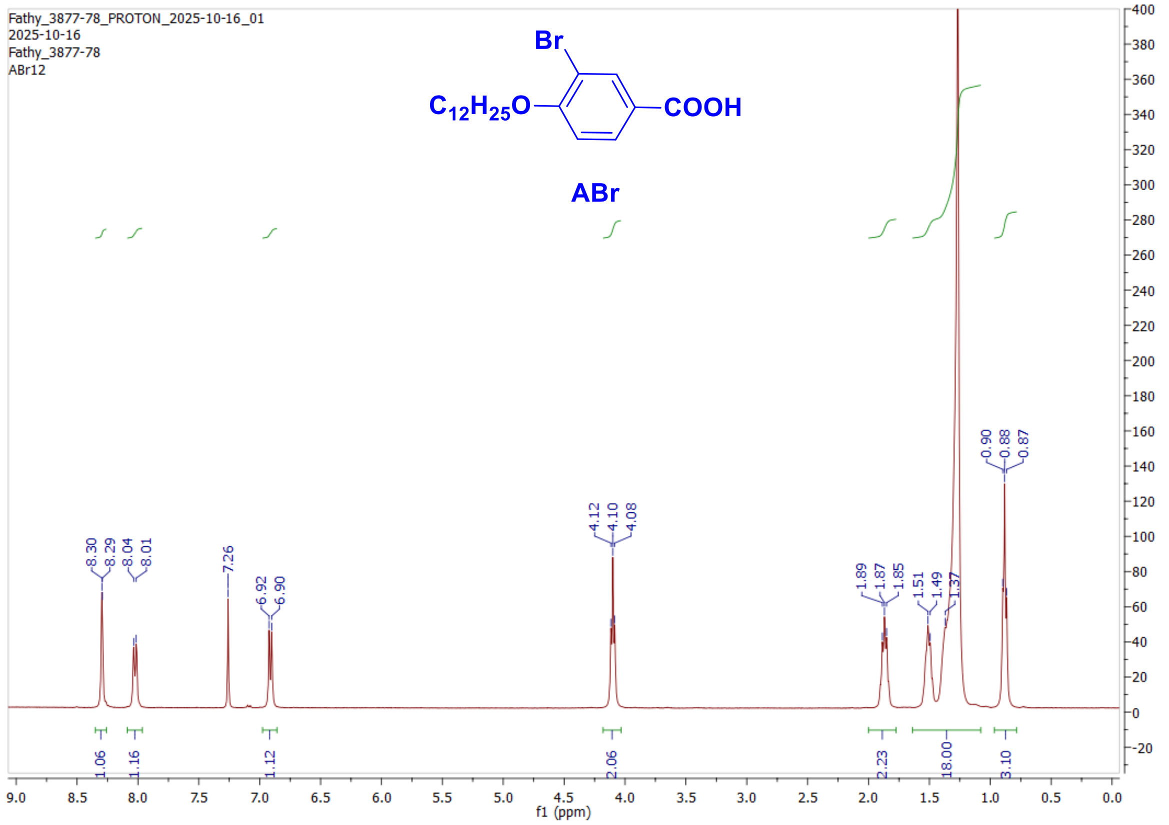

Figure S3a. ^{1}H NMR of compound **ABr**

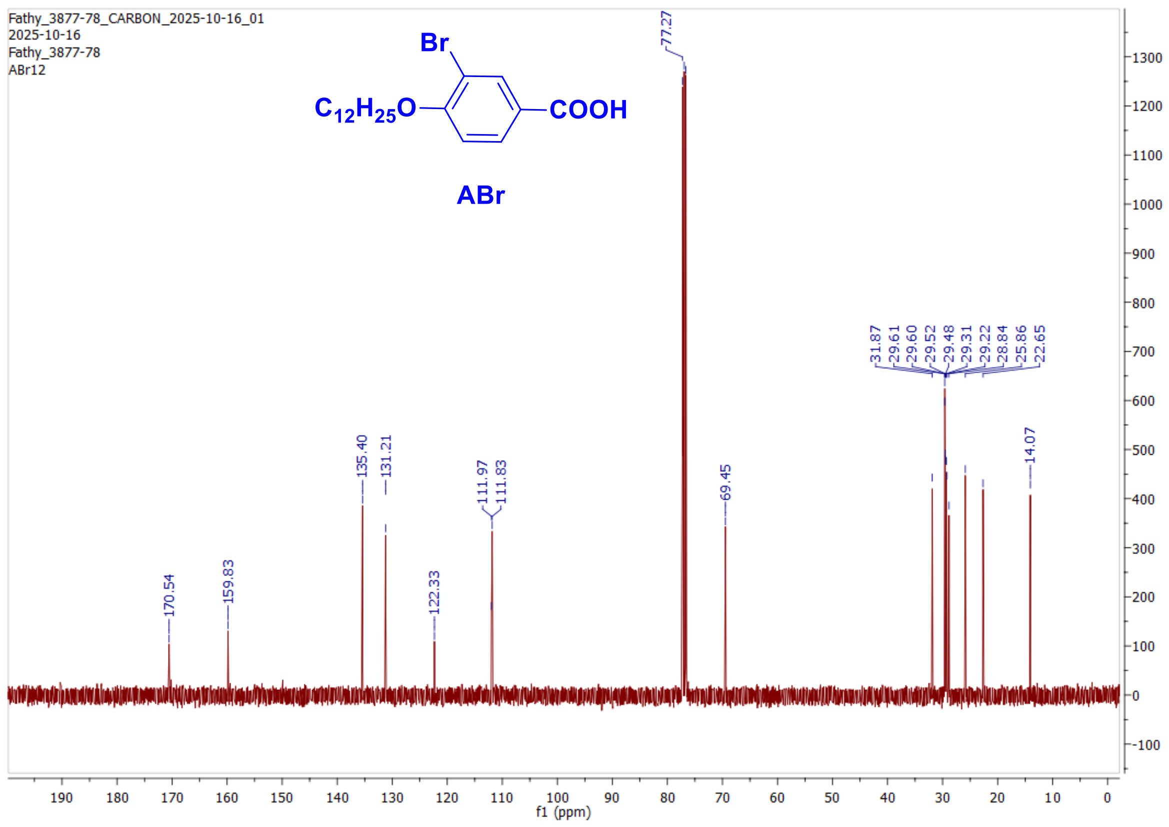

Figure S3b. ^{13}C NMR of compound **ABr**

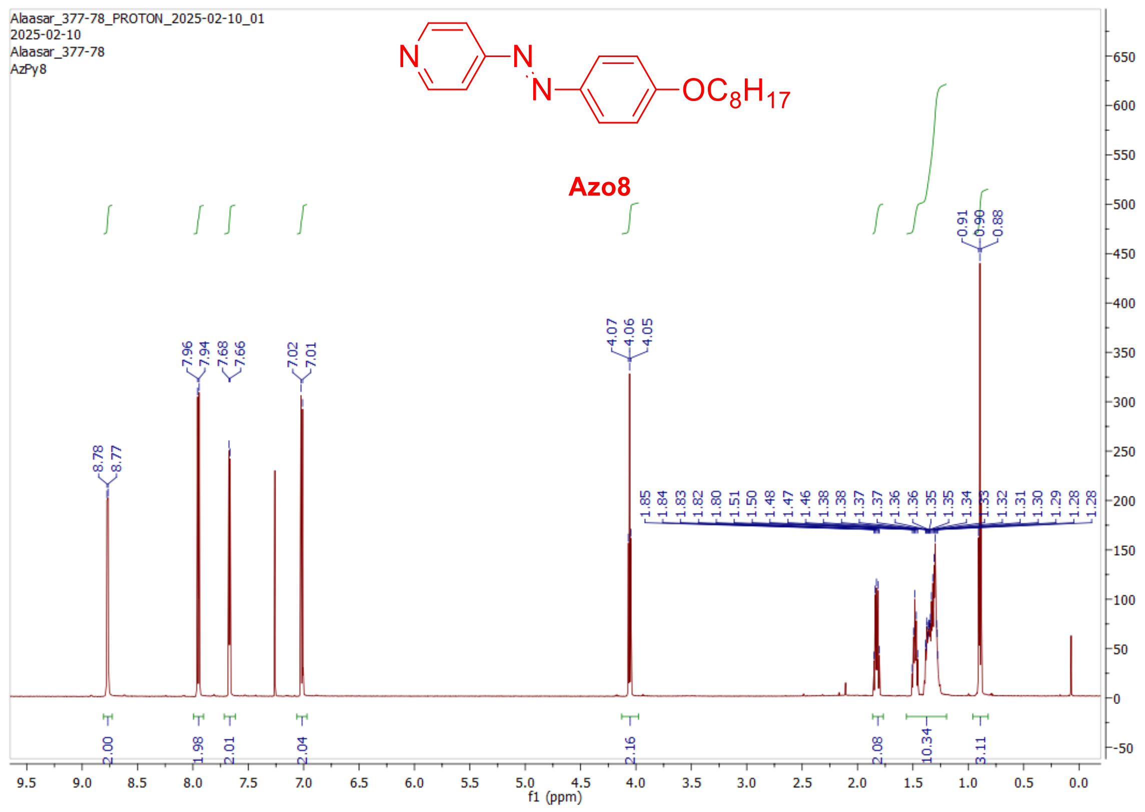

Figure S4a. ^{1}H NMR of compound **Azo8**

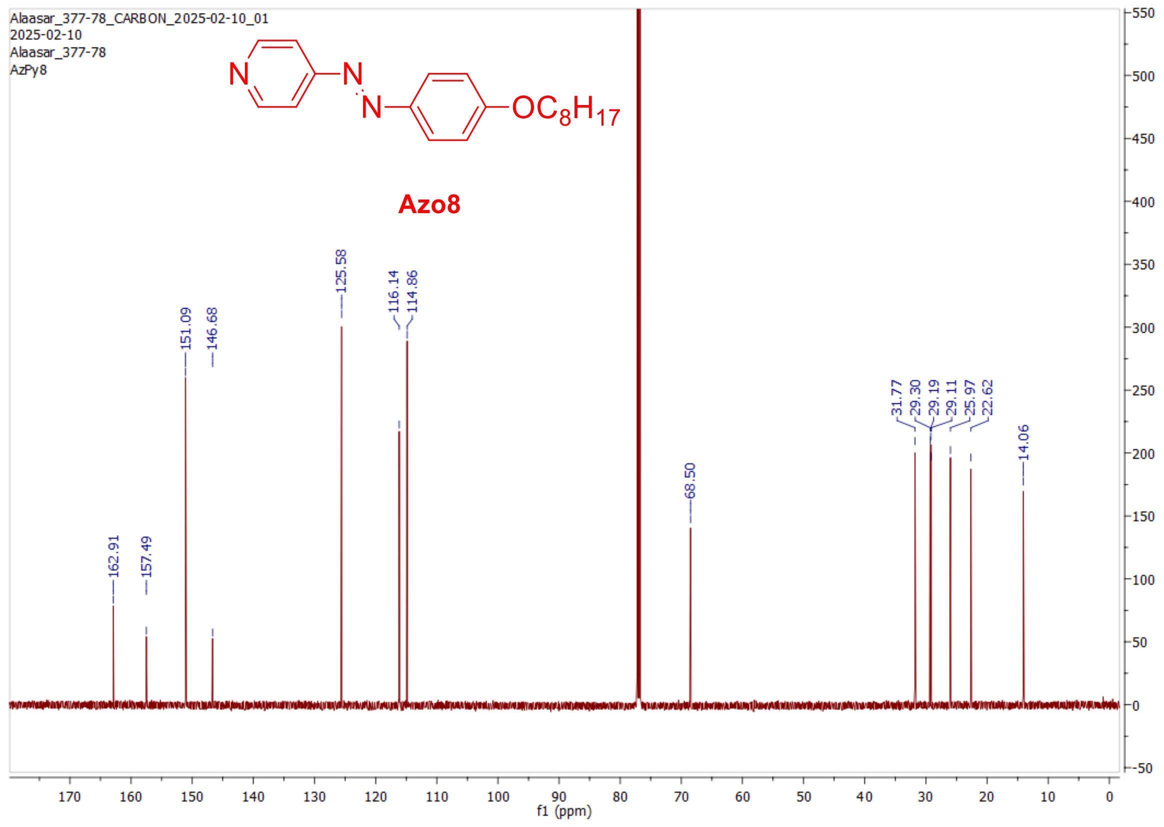

Figure S4b. ^{13}C NMR of compound **Azo8**

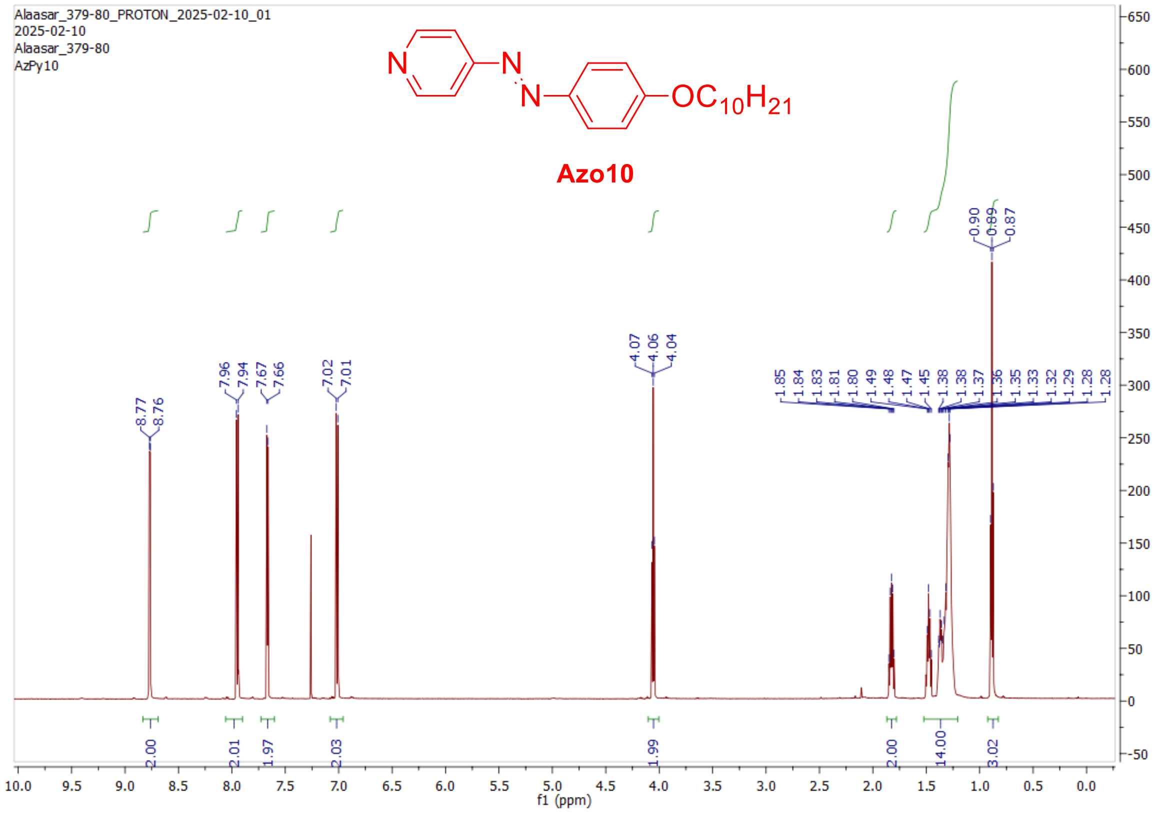

Figure S5a. ^{1}H NMR of compound **Azo10**

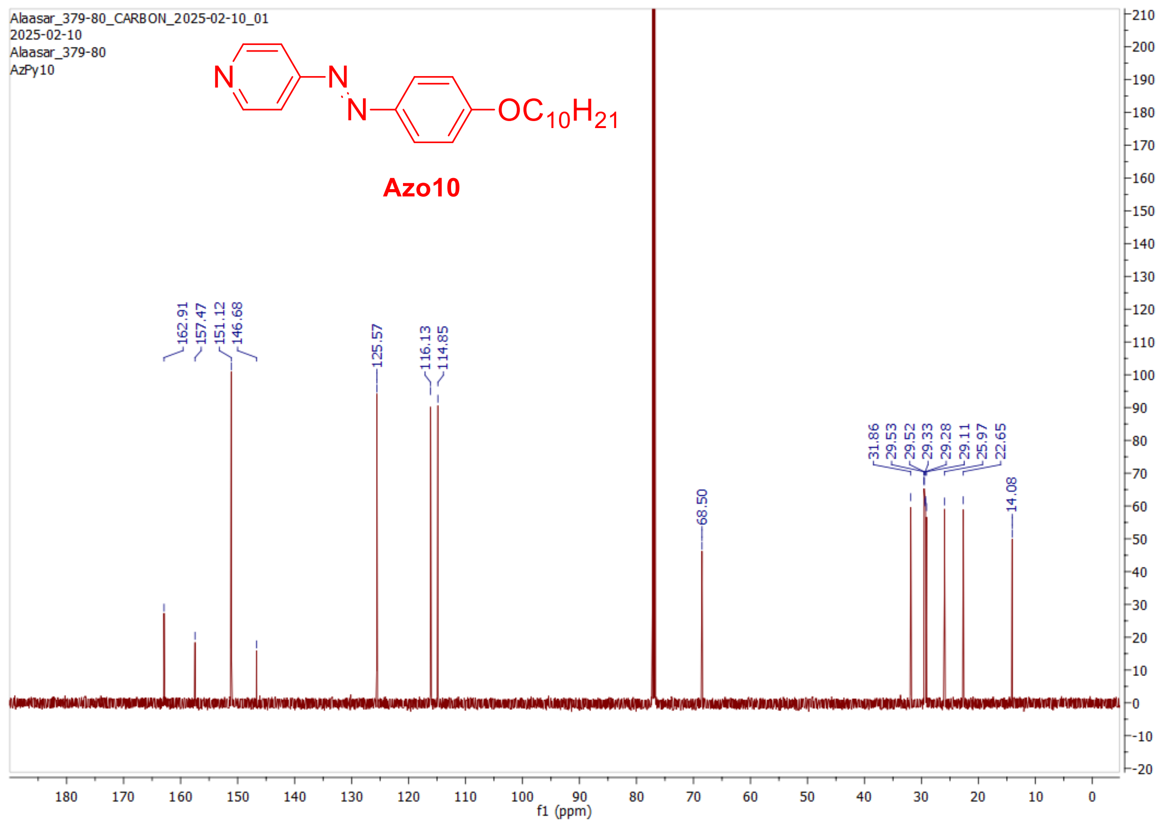

Figure S5b. ^{13}C NMR of compound **Azo10**

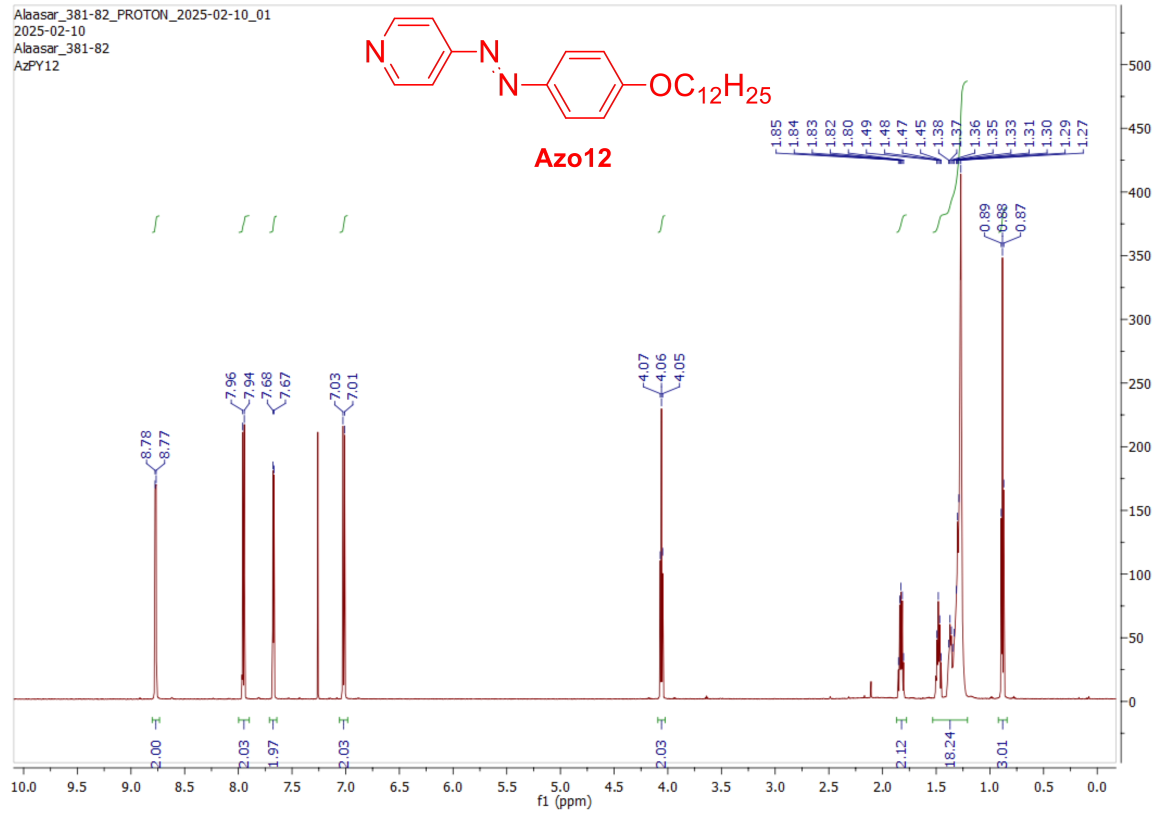

Figure S6a. ^{1}H NMR of compound **Azo12**

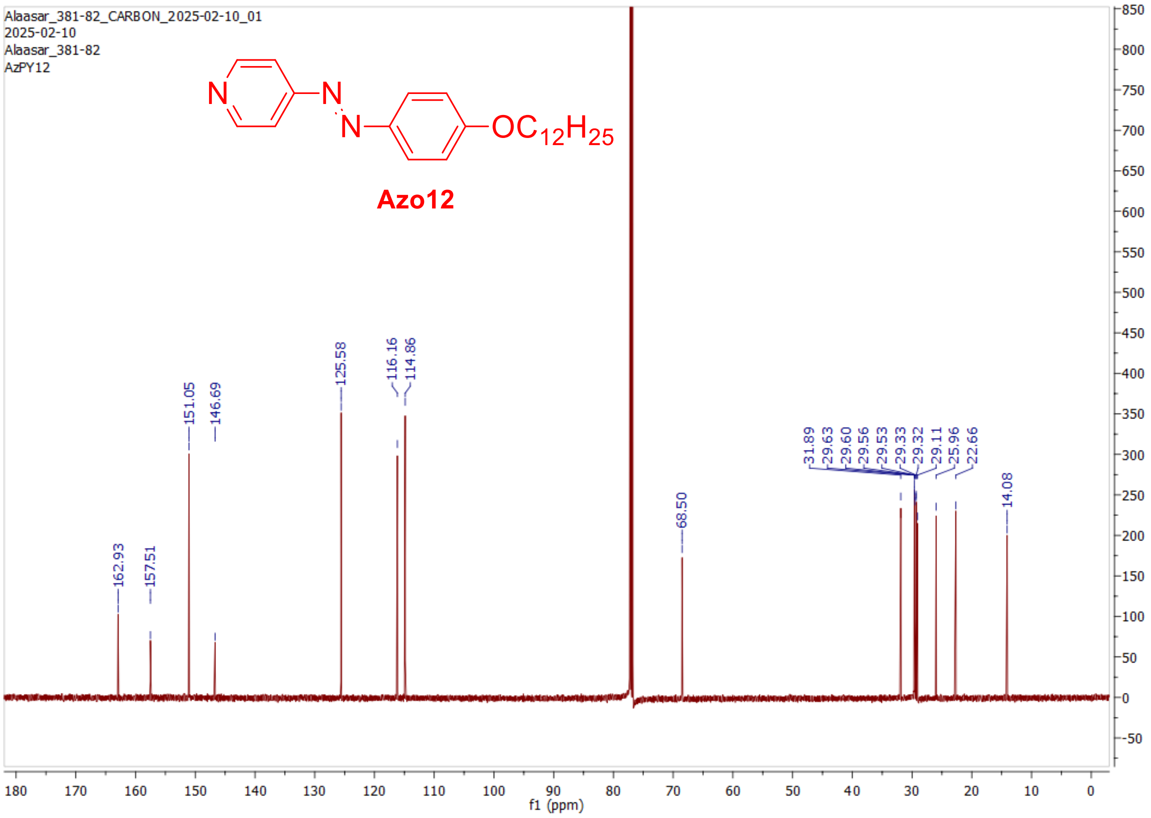

Figure S6b. ^{13}C NMR of compound **Azo12**

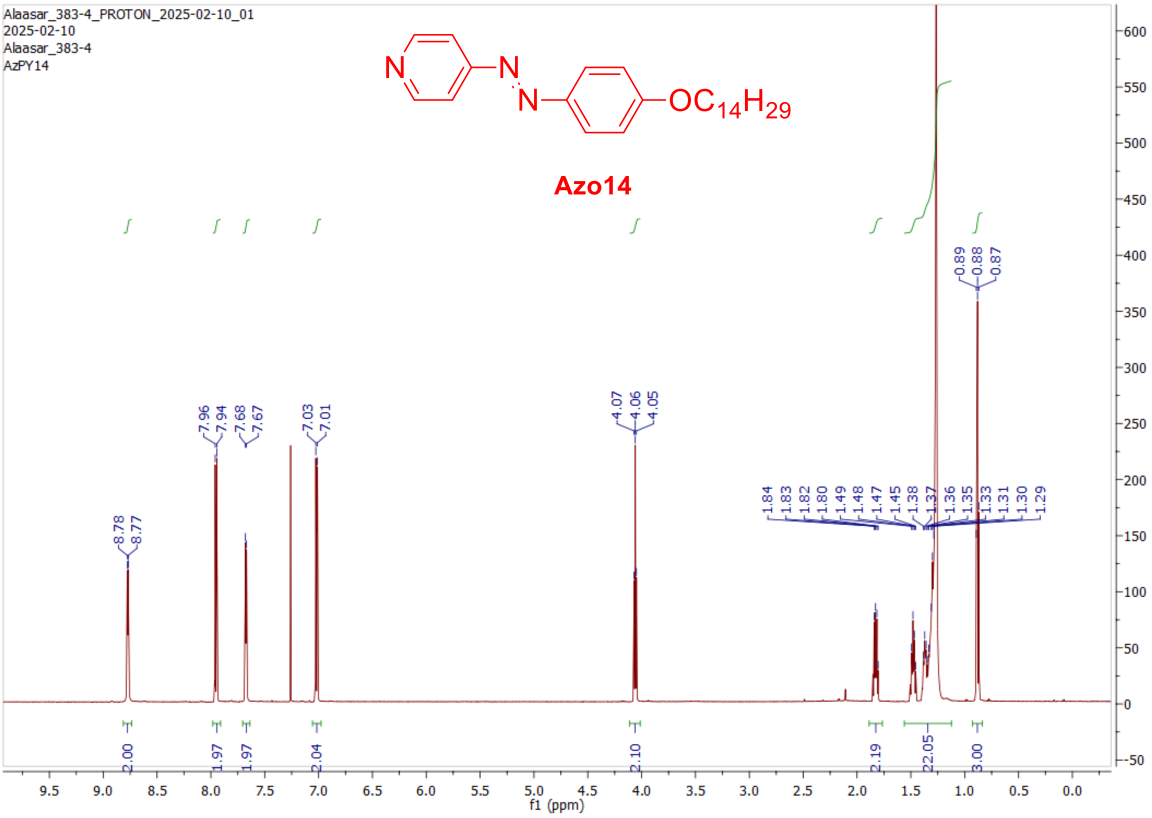

Figure S7a. ^{1}H NMR of compound **Azo14**

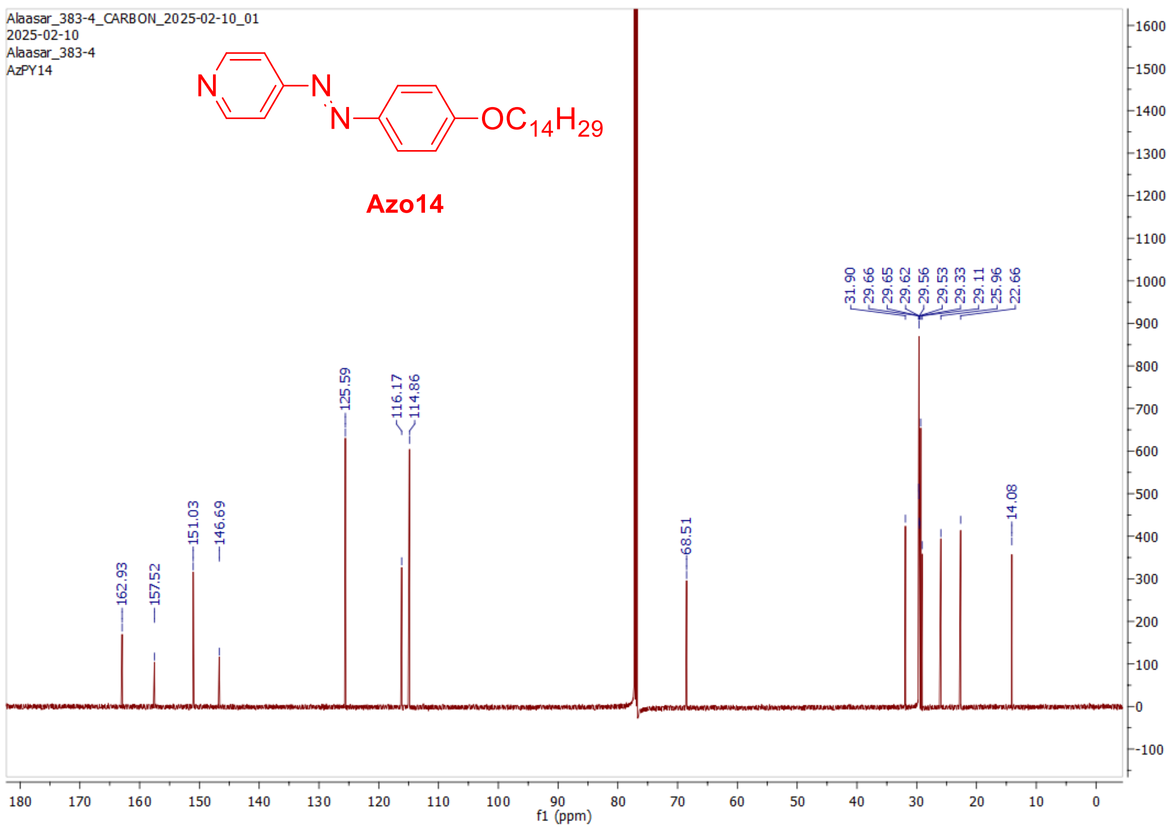

Figure S7b. ^{13}C NMR of compound **Azo14**

2. Additional Data

2.1. Additional IR-data

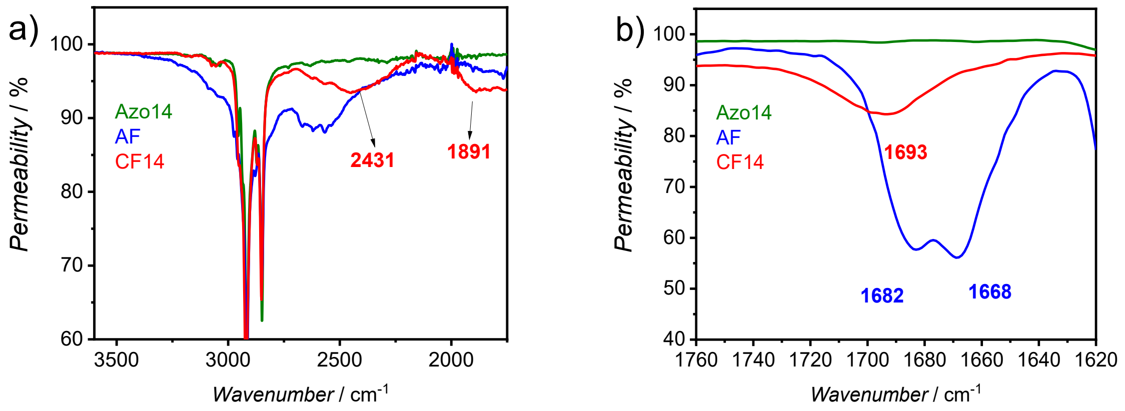

Figure S8. FTIR spectra of the supramolecule **CF14** (red) and its complementary components **AF** (blue), **Azo14** (green) in the crystalline state at room temperature: a) enlarged area between 1750 cm^{-1} and 3600 cm^{-1}, and b) between 1620 cm^{-1} and 1760 cm^{-1}.

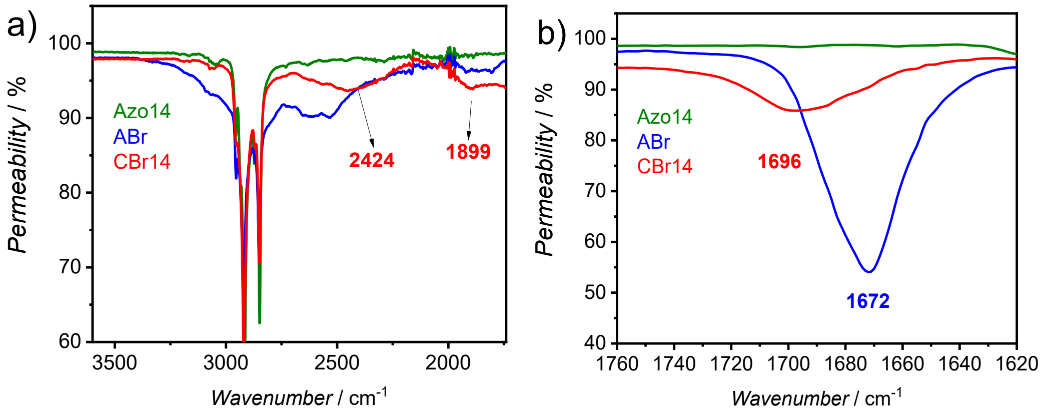

Figure S9. FTIR spectra of the supramolecule **CBr14** (red) and its complementary components **ABr** (blue), **Azo14** (green) in the crystalline state at room temperature: a) enlarged area between 1750 cm^{-1} and 3600 cm^{-1}, and b) between 1620 cm^{-1} and 1760 cm^{-1}.

2.2. Additional DSC traces

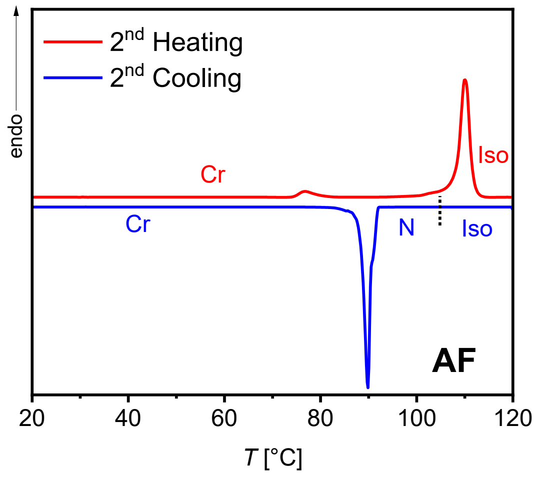

Figure S10. DSC heating and cooling traces of the 3-fluoro-4-dodecyloxybenzoic acid (**AF**) recorded at 10 K min^{-1}.

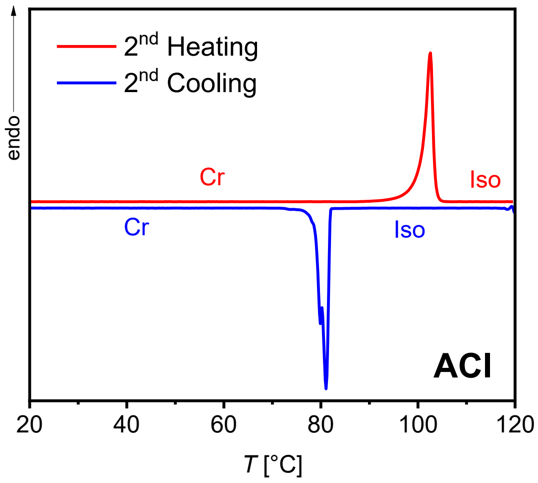

Figure S11. DSC heating and cooling traces of the 3-chloro-4-dodecyloxybenzoic acid (**ACl**) recorded at 10 K min^{-1}.

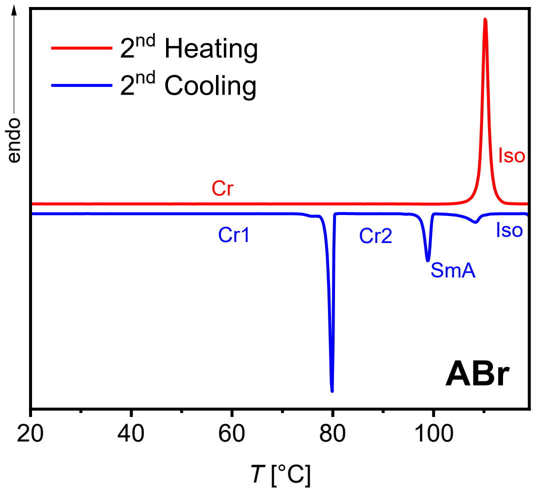

Figure S12. DSC heating and cooling traces of the 3-bromo-4-dodecyloxybenzoic acid (**ABr**) recorded at 10 K min^{-1}.

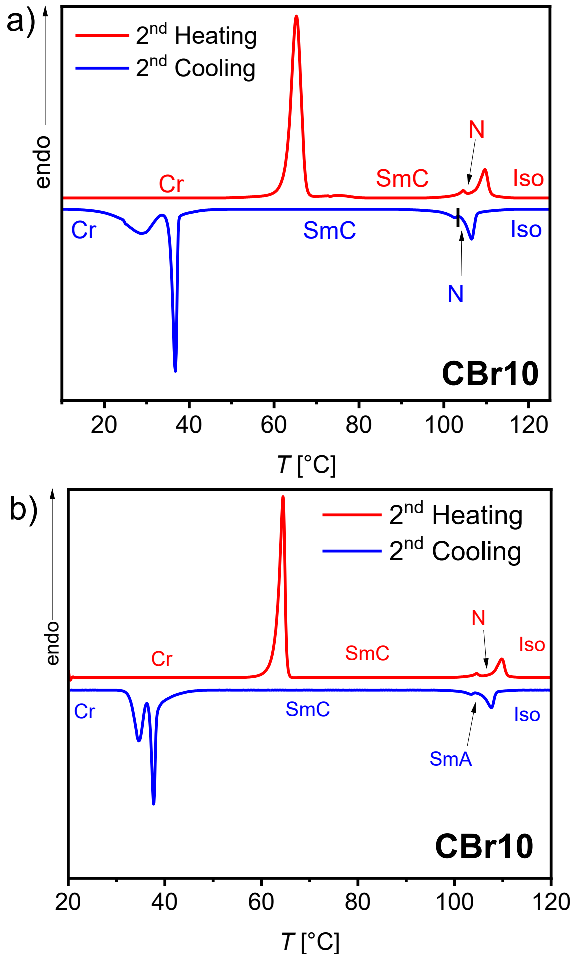

Figure S13. DSC heating and cooling traces of the supramolecule **CBr10** recorded at: a) 10 K min^{-1} and a) 5 K min^{-1}.

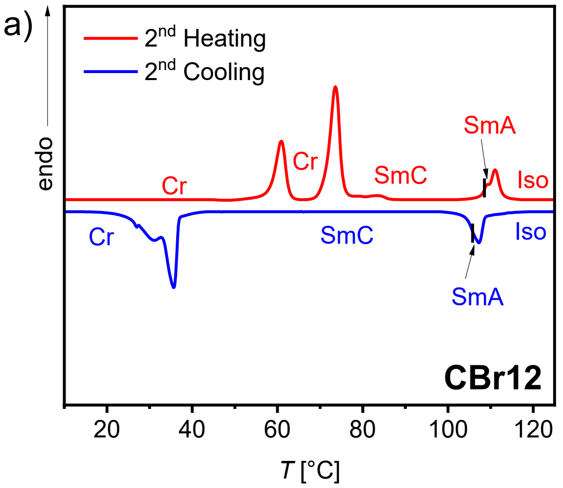

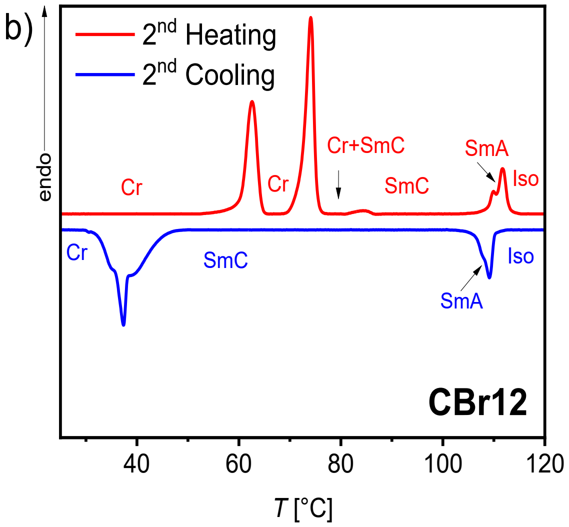

Figure S14. DSC heating and cooling traces of the supramolecule **CBr12** recorded at: a) 10 K min^{-1} and b) 5 K min^{-1}.

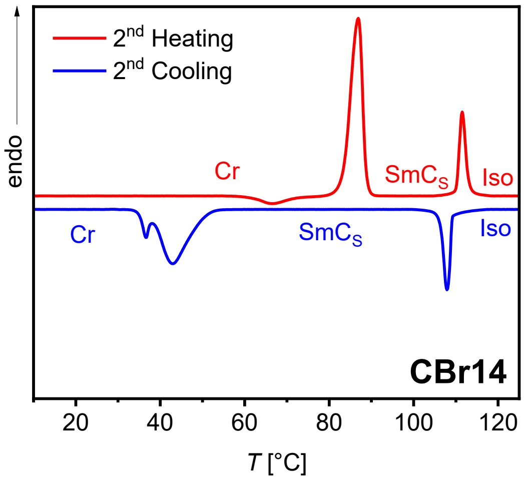

Figure S15. DSC heating and cooling traces of the supramolecule **CBr14** recorded at 10 K min^{-1}.

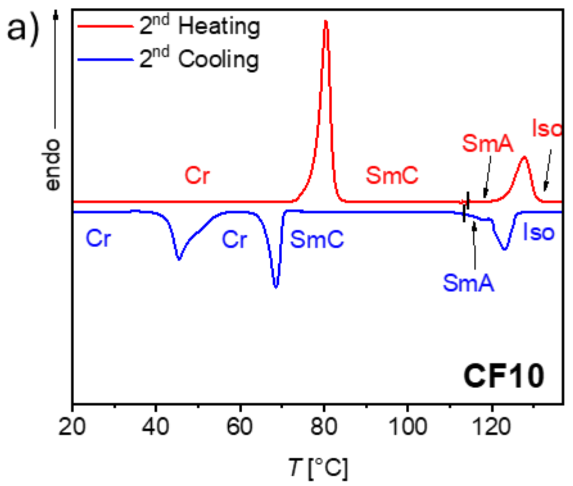

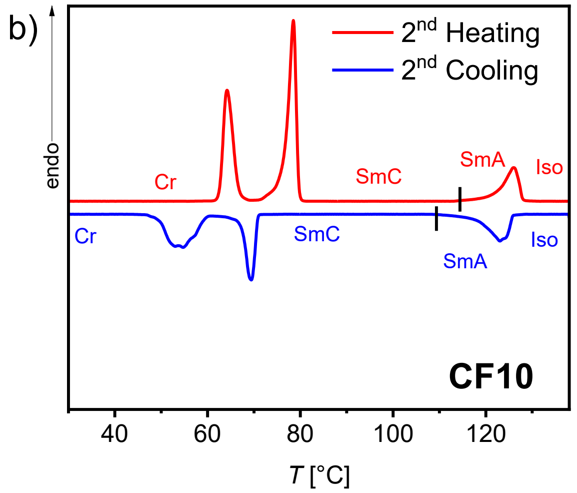

Figure S16. DSC heating and cooling traces of the supramolecule **CF10** recorded at: a) 10 K min^{-1} and b) 5 K min^{-1}.

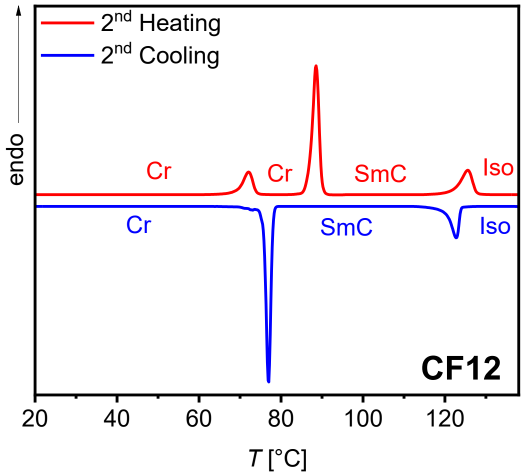

Figure S17. DSC heating and cooling traces of the supramolecule **CF12** recorded at 10 K min^{-1}.

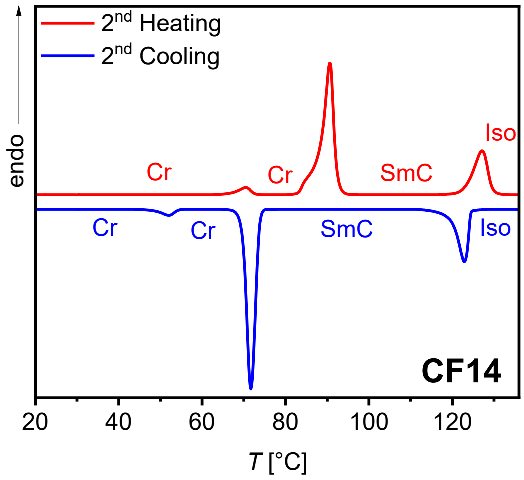

Figure S18. DSC heating and cooling traces of the supramolecule **CF14** recorded at 10 K min^{-1}.

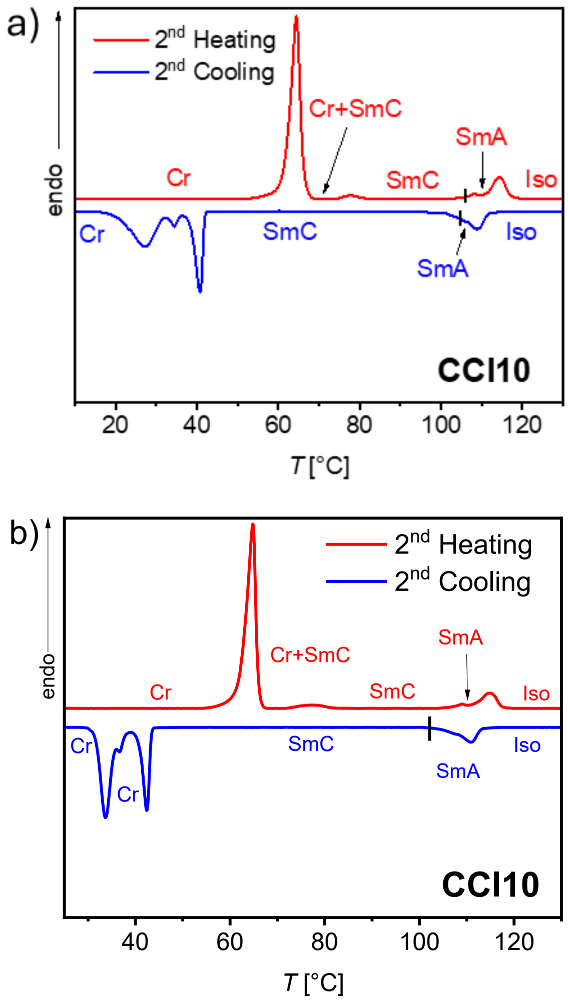

Figure S19. DSC heating and cooling traces of the supramolecule **CCl10** recorded at: a) 10 K min^{-1} and b) 5 K min^{-1}.

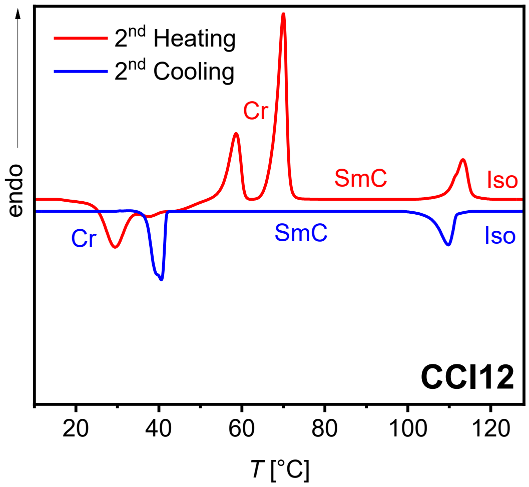

Figure S20. DSC heating and cooling traces of the supramolecule **CCl12** recorded at 10 K min^{-1}.

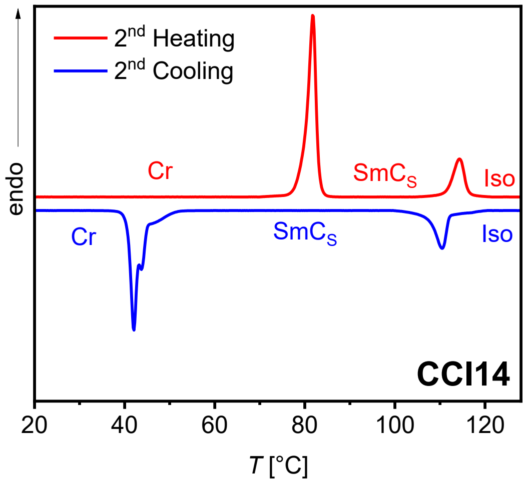

Figure S21. DSC heating and cooling traces of the supramolecule **CCl14** recorded at 10 K min^{-1}.

2.3. *Additional Textures*

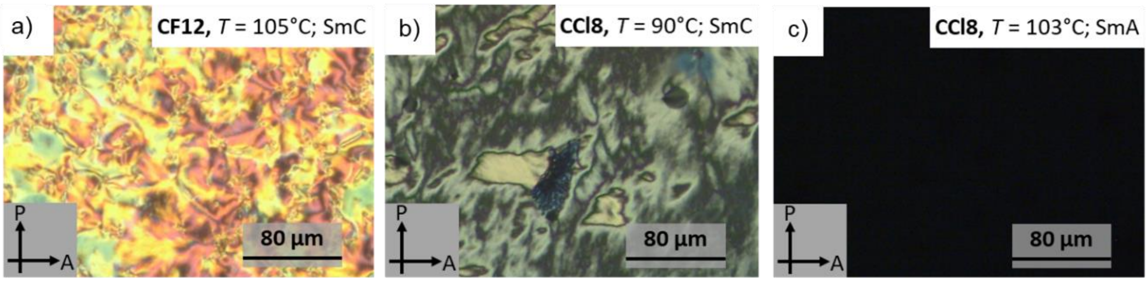

Figure S22. Optical textures observed on cooling of the supramolecules: a) **CF12** in the SmC phase at T = 105°C, and **CCl8** b) in the SmC phase at T = 80°C, and c) in the SmA phase at T = 103°C.

3. XRD data

3.1. *SAXS patterns*

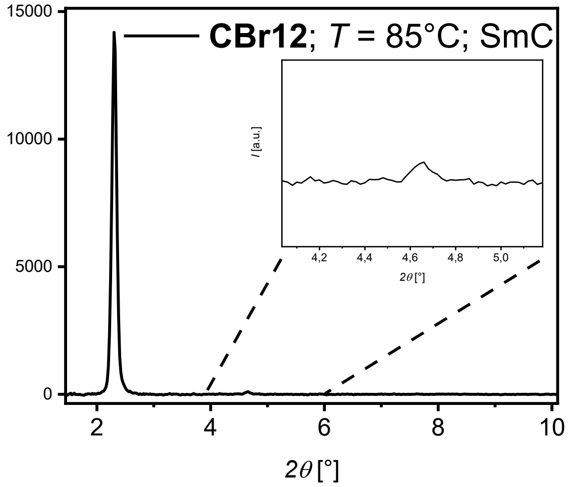

Figure S23. SAXS pattern recorded on cooling with a cooling rate of 10K/min of the supramolecule **CBr12** in the SmC_s phase.

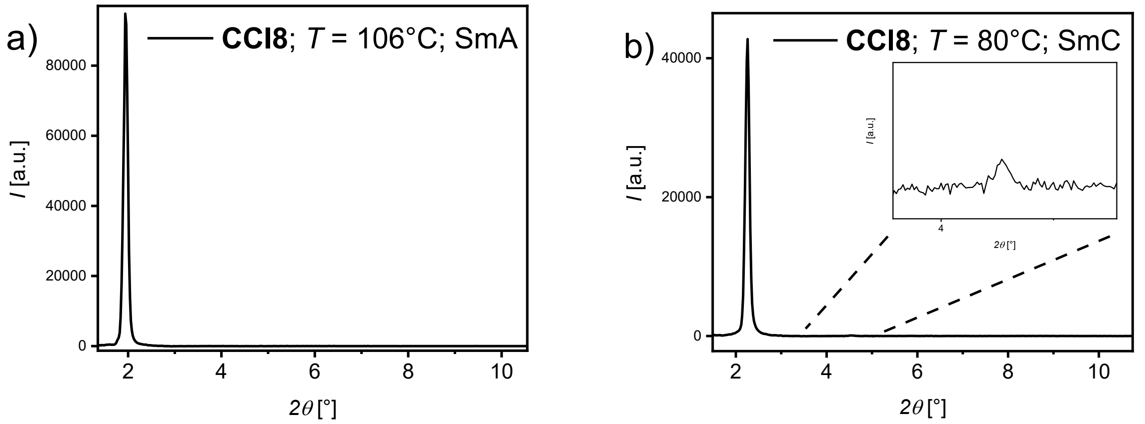

Figure S24. SAXS pattern recorded on cooling with a cooling rate of 10K/min of the supramolecule **CCl8** at the indicated temperatures; a) in the SmA phase and b) in the SmC phase.

3.2. *WAXS patterns*

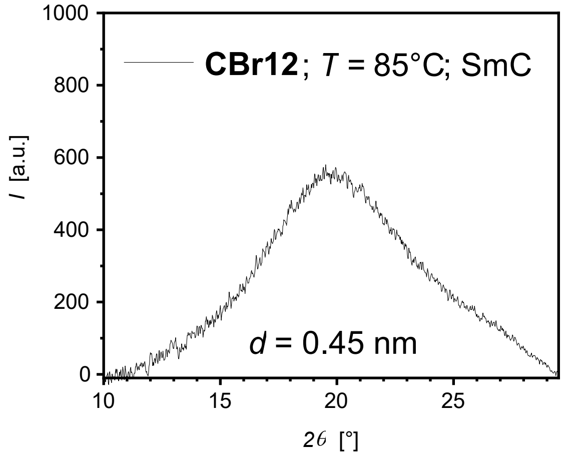

Figure S25. WAXS pattern recorded on cooling with a cooling rate of 10K/min of the supramolecule **CBr12** in the SmC phase.

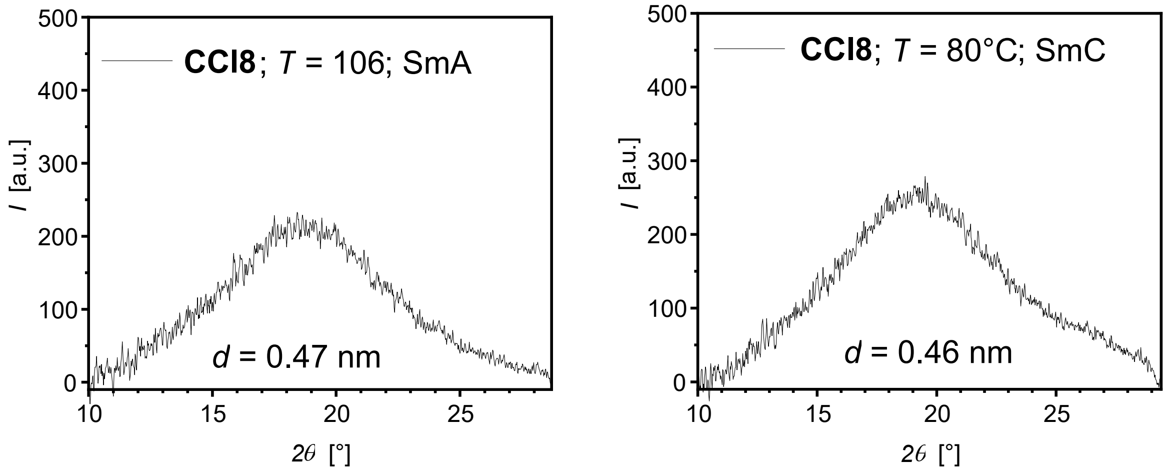

Figure S26. WAXS pattern recorded on cooling with a cooling rate of 10K/min of the supramolecule **CCl8** at the indicated temperatures; a) in the SmA phase and b) in the SmC phase.

2D-WAXS Pattern

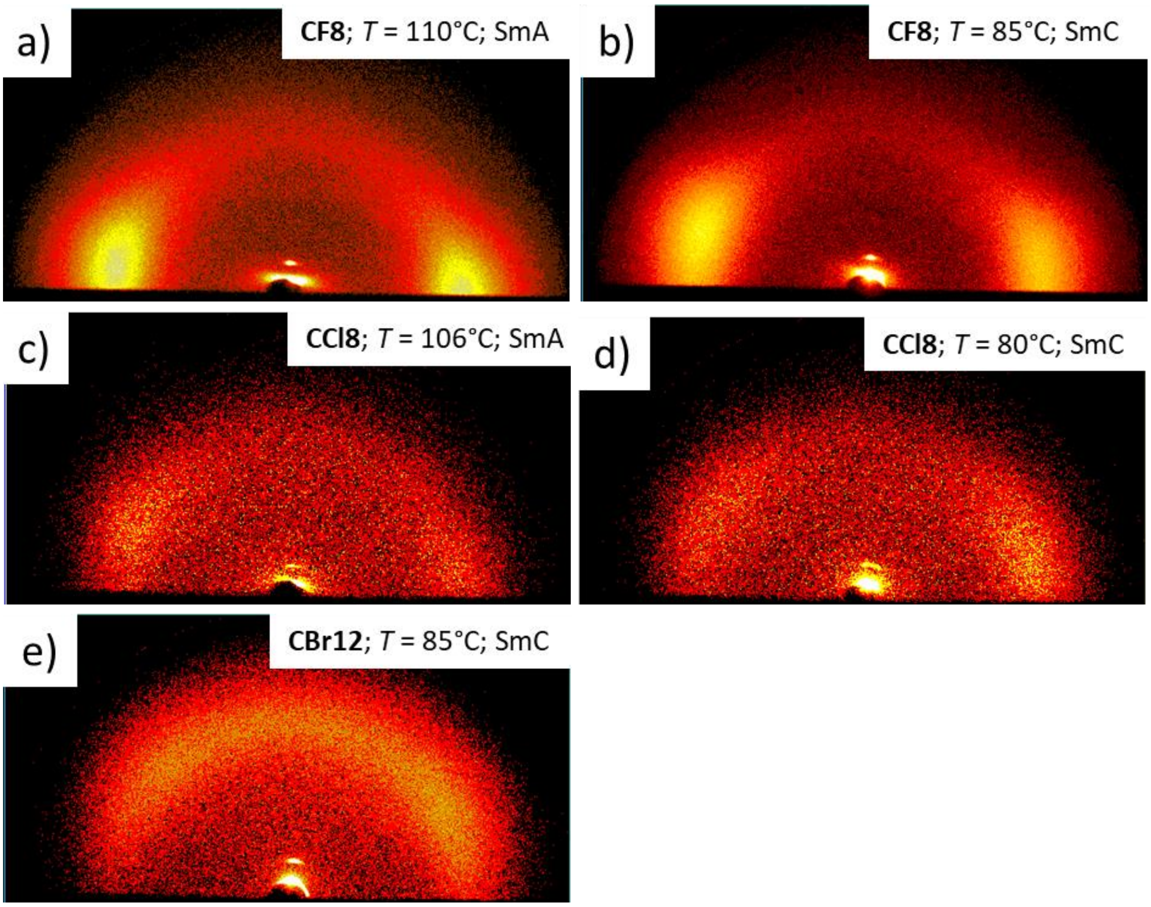

Figure S27. 2D-WAXS pattern recorded on cooling with a rate of 10K/min in different LC phases at the indicated temperatures.

3.3. *Structural data*

Table S1. Numerical SAXS data of the supramolecule **CBr12** in the SmC_s phase at 85 °C.

2θ [°]	$d_{obs.}$ [nm]	$d_{calc.}$ [nm]	Δ	hkl
2.305	3.833	3.833	0.00	10
4.651	1.900	1.916	0.02	20
20.023	0.443			

Table S2. Numerical SAXS data of the supramolecule **CF8** in the SmA phase at 110 °C.

2θ [°]	$d_{obs.}$ [nm]	$d_{calc.}$ [nm]	Δ	hkl
1.947	4.537	4.537	0.00	10
3.903	2.264	2.269	0.00	20
18.920	0.469			

Table S3. Numerical SAXS data of the supramolecule **CF8** in the SmC_s phase at 80 °C.

2θ [°]		$d_{obs.}$ [nm]	$d_{calc.}$ [nm]	Δ	hkl
2.068		4.272	4.272	0.00	10
4.149		2.130	2.136	0.01	20
19.241		0.461			

Table S4. Numerical SAXS data of the supramolecule **CCl8** in the SmA phase at 106 °C.

2θ [°]	$d_{obs.}$ [nm]	$d_{calc.}$ [nm]	Δ	hkl
1.950	4.530	4.530	0.00	10
19.197	0.462			

Table S5. Numerical SAXS data of the supramolecule **CCl8** in the SmC_s phase at 80 °C.

2θ [°]	$d_{obs.}$ [nm]	$d_{calc.}$ [nm]	Δ	hkl
2.255	3.918	3.918	0.00	10
4.546	1.944	1.959	0.02	20
18.740	0.473			

4. References

1 S. M. Kelly, *Helv. Chim. Acta*, 1989, **72**, 594–607.

2 N. Trišović, J. Antanasijević, J. Rogan, D. Poleti, T. Tóth-Katona, M. Salamonczyk, A. Jákli and K. Fodor-Csorba, *New J. Chem.*, 2016, **40**, 6977–6985.
